# Phenotypic characterization of drought responses in red clover (*Trifolium pratense* L.)

**DOI:** 10.3389/fpls.2023.1304411

**Published:** 2024-01-12

**Authors:** Tim Vleugels, Aamir Saleem, Reena Dubey, Hilde Muylle, Irene Borra-Serrano, Peter Lootens, Tom De Swaef, Isabel Roldán-Ruiz

**Affiliations:** ILVO (Flanders Research Institute for Agriculture, Fisheries and Food), Plant Sciences Unit, Melle, Belgium

**Keywords:** *Trifolium pratense*, drought responses, phenotyping, UAV, canopy temperature, HTFP

## Abstract

**Introduction:**

Red clover (*Trifolium pratense*) is a protein-rich, short-lived perennial forage crop that can achieve high yields, but suffers increasingly from drought in different cultivation areas. Breeding for increased adaptation to drought is becoming essential, but at this stage it is unclear which traits breeders should target to phenotype responses to drought that allow them to identify the most promising red clover genotypes. In this study, we assessed how prolonged periods of drought affected plant growth in field conditions, and which traits could be used to distinguish better adapted plant material.

**Methods:**

A diverse panel of 395 red clover accessions was evaluated during two growing seasons. We simulated 6-to-8-week drought periods during two consecutive summers, using mobile rain-out shelters, while an irrigated control field was established in an adjacent parcel. Plant growth was monitored throughout both growing seasons using multiple flights with a drone equipped with RGB and thermal sensors. At various observation moments throughout both growing seasons, we measured canopy cover (CC) and canopy height (CH). The crop water stress index (CWSI) was determined at two moments, during or shortly after the drought event.

**Results:**

Manual and UAV-derived measurements for CH were well correlated, indicating that UAV-derived measurements can be reliably used in red clover. In both years, CC, CH and CWSI were affected by drought, with measurable growth reductions by the end of the drought periods, and during the recovery phase. We found that the end of the drought treatment and the recovery phase of approximately 20 days after drought were suitable periods to phenotype drought responses and to distinguish among genotypes.

**Discussion:**

Multifactorial analysis of accession responses revealed interactions of the maturity type with drought responses, which suggests the presence of two independent strategies in red clover: ‘drought tolerance’ and ‘drought recovery’. We further found that a large proportion of the accessions able to perform well under well-watered conditions were also the ones that were less affected by drought. The results of this investigation are interpreted in view of the development of breeding for adaptation to drought in red clover.

## Introduction

Red clover (*Trifolium pratense* L.) is an important forage legume crop in temperate regions around the world (Annicchiarico and Pagnotta, 2012). It is either grown in mixtures with grasses or as a monoculture (Taylor and Quesenberry, 1996). Farmers appreciate red clover for its capacity to fix atmospheric nitrogen in symbiosis with soil bacteria, its high forage yield and crude protein content, and its high palatability (Kjærgaard, 2003). Red clover is a short-lived perennial that remains productive for about three years, after which plants start to degenerate. Plants develop a deep, well-branched taproot during the first year after sowing, that is later complemented with adventitious roots (Taylor and Quesenberry, 1996). Commercial varieties are usually classified into two maturity types (Boucher, 2016), as this has important implications for their agronomic use. ‘Double-cut’ varieties develop early in spring, have a strong regrowth capacity, a higher shoot/root ratio, and keep growing until late in autumn if environmental conditions are favorable (Hejduk and Knot, 2010). ‘Single-cut’ varieties initiate growth later in spring, display slower re-growth capacity after mowing, and exhibit earlier autumn dormancy - traits that render them more persistent and winter-hardy (Hejduk and Knot, 2010). Single-cut varieties require longer photoperiods than double-cut varieties to initiate flowering (day lengths of 14 h or more), and rarely flower in-between mowing events (Taylor and Quesenberry, 1996; Boucher, 2016). Single-cut types are commonly grown in Nordic climates, while the double-cut types are better adapted to temperate and warm climates (Taylor and Quesenberry, 1996).

In the light of climate change, it is crucial that breeders anticipate and create new red clover varieties that can cope with periods of drought (Boucher, 2016). While red clover has been traditionally regarded as a fairly drought-tolerant crop when compared to other forages (Annicchiarico et al., 2015), changing climatic conditions in Europe are expected to bring about warmer summers with longer periods of drought and less rainfall (IPCC, 2023). Consequently, drought is expected to decrease productivity in European forage crops (Dellar et al., 2018), possibly also in red clover. Indeed, some red clover varieties, such as those selected for cultivation in temperate and Nordic climates, might display a high level of drought susceptibility.

Investigating the response of crops to drought is however not simple. Plants can express different mechanisms to cope with drought (Fang and Xiong, 2015), and according to Aslam et al. (2015) at least four responses can be distinguished: (1) drought escape, which prevents exposure to terminal drought stress through earliness; (2) drought avoidance, which implies increasing water uptake or reducing water losses during periods of drought; (3) drought tolerance, which maintains physiological processes during drought stress and allows to conserve a certain degree of productivity during drought; and (4) quick drought recovery, which represents the speed at which plant productivity resumes to a normal level after drought. In forage crops, mainly ‘drought tolerance’ and ‘drought recovery’ have been described (Pembleton and Sathish, 2014) but few studies have investigated specifically the mechanisms active in red clover. For example, in the related legume crop alfalfa (*Medicago sativa* L.), drought tolerance has been associated with the capacity to limit water losses through the reduction of transpiration rates or the reduction of leaf size and aboveground biomass, and with the capacity to absorb more water, through the formation of more lateral roots (Hanson et al., 2015; Quan et al., 2016). Drought-tolerant plants protect cell membranes from oxidative damage, and display higher antioxidant enzyme activity during stress (Quan et al., 2016). In both alfalfa and red clover, drought recovery has been associated with the ability to maintain viable tissues in the crown and with the availability of metabolites to support re-growth (Pembleton et al., 2010; Loucks et al., 2018). In these perennial crops, drought stress reduces the number and size of viable crown buds, and lowers starch concentrations in the taproot, both of which are essential for regrowth after a period of drought (Pembleton et al., 2010).

An important prerequisite for red cover breeders to identify genotypes adapted to drought conditions is an evaluation of the variation available in breeding germplasm and the definition of effective selection criteria. However, the response of red clover to drought has not been thoroughly characterized yet. Available publications either describe work performed in growth chambers using only a few accessions (Loucks et al., 2018), report the evaluation of accessions of narrow geographical ranges (Annicchiarico and Pagnotta, 2012), or focus on the molecular mechanisms associated with some responses to drought, such as the abundance of stress-related proteins (Vaseva et al., 2011) or changes in the transcriptome (Yates et al., 2014). To our knowledge, no prior studies have described the response to drought of a large collection of red clover genotypes in terms of growth and productivity, nor have they proposed specific easy-measurable traits that can be used by breeders. In breeding context, phenotyping drought responses is preferably done in the field, and rain-out shelters that block the precipitation, while having minimal effects on temperature and light conditions are ideal tools (Parra et al., 2012; De Swaef et al., 2021). In temperate climates, the most relevant period to evaluate the drought response of red clover is early summer, right after the first cut. In this period, soil moisture reserves built up during the winter can already be depleted, and the plants rely mostly on precipitation for growth. Furthermore, an optimal re-growth in this period is essential to obtain a good yearly productivity. All these aspects should be considered when designing relevant screening experiments.

The present work was performed in the framework of the EUCLEG project (Horizon 2020 Programme for Research and Innovation). A strategic goal of EUCLEG was to develop efficient breeding strategies in five legume crops, among which red clover, by analyzing key agronomic and quality traits. Here we present the work performed to characterize for the first time the diversity of responses to drought available in a collection of 395 red clover accessions from 14 countries. Field-grown plants were subjected to two periods of drought in early summer during two production years. Forage crop breeding relies mostly on visual observations and manual measurements, but high-throughput field phenotyping (HTFP) using unmanned aerial vehicles (UAV) equipped with different imaging sensors are a promising alternative, especially when dealing with large numbers of plants or field plots (Araus and Cairns, 2014; Borra-Serrano et al., 2019). An additional advantage of UAV measurements is that objective, repeated measurements can be made over a course of time to follow-up plant growth in detail over an entire growing season. While, to the best of our knowledge, the use of indices derived from RGB or thermal sensors mounted on an UAV has not yet been explored in red clover, they have proven very useful to determine the drought responses of many other crops such as forage grasses (De Swaef et al., 2021), alfalfa (Surault et al., 2021), and soybean (Saleem et al., 2022).

The objectives of our study were: (i) to characterize for the first time the responses to drought of a diverse panel of red clover accessions; (ii) to appraise the use of UAV-derived measurements in characterizing growth and drought responses in red clover; (iii) to assess the “broad-sense” heritability of traits related to drought responses in this diverse collection, (iv) to assess whether the response of red clover to drought differs between the first and the second year of production, and (v) to identify traits that can be targeted by breeders to improve adaptation to drought in European red clover.

## Materials and methods

### Experimental design

The plant materials used in this study have been described by Frey et al. (2022). Here we used a subset of 395 red clover accessions of diverse geographical origin and including different population types: ecotypes, landraces, cultivars and breeding material from 14 countries (**Supplementary Table S1**).

**Figure 1:**
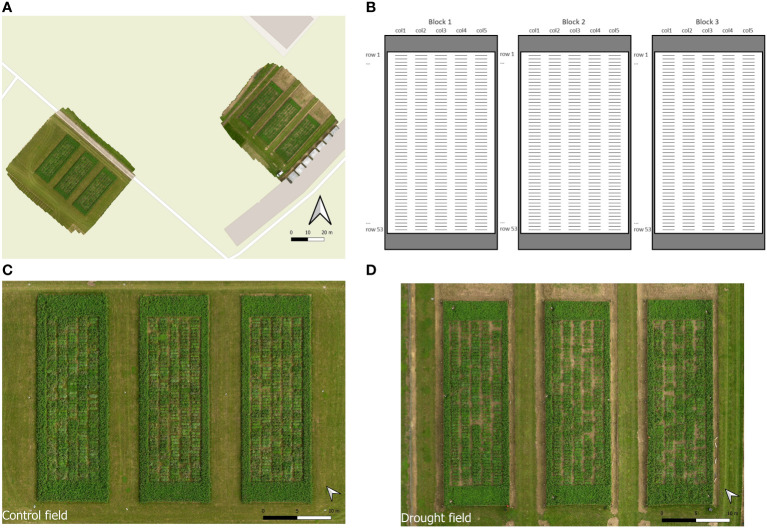
Overview of the trial with **(A)** a UAV-derived picture of the control (left) and drought (right) fields, **(B)** a schematic overview of the organization of the fields with blocks, columns and rows indicated, and UAV-derived photographs of the control **(C)** and drought **(D)** fields.

Our trial comprised two adjacent fields of sandy loam soil, which were sown in August 2018 in Melle, Belgium (51.00° N, 3.80° E) (**Figure 1A**). One of these fields, further named the ‘control field’, was irrigated and served as control. The other field, further named the ‘drought field’, was subjected to drought using mobile rain-out shelters as described below. Both fields were established following an identical, incomplete block design with two randomized repetitions of 395 accessions, distributed over three blocks, with each rain-out shelter representing one block (**Figure 1**). Each block measured 10 m x 30 m and contained five strips (further named columns) with 53 individual plots (further named rows), spaced 45 cm apart. Each plot represented a single accession sown in a 1 m line. All accessions were replicated twice over the three blocks, except the control cultivar ‘Lemmon’ which was replicated seven times to prevent empty entries in the design. The control and drought fields each contained a total of 795 plots (3 blocks x 5 columns x 53 rows). Around each block, border strips containing ‘Lemmon’ were sown to reduce border effects. The control and drought fields were treated identically in terms of sowing, mowing regime, fertilization, weed control and most observations.

In the drought field, the perennial crop was subjected to early summer drought in two consecutive years, to evaluate the response to drought of plants in different stages of development, i.e. plants in the first production year, which do not yet have a fully developed root system, and the same plants in the second production year, which have a more developed root system but are also older. The two trial years cannot be considered as replicates, as the developmental status of the perennial crop was different.

The maturity type of each accession was not known in advance, but it was possible to identify the accessions’ maturity using flowering data recorded during the first production year. For each accession, we had data available from four plots in our trial: two control plots and two drought plots. We classified an accession as ‘early flowering’ when it flowered in at least one plot – representing mostly double-cut types, and ‘late flowering’ when it failed to flower in any of the four plots – representing mostly single-cut types.

### Agronomic management

The trial was sown manually at 1 cm depth on a finely prepared seed bed. Each plot was sown with 90 germinable seeds. The number of actually sown seeds per plot depended on the germination of each accession seed lot, which was provided by the EUCLEG consortium: 90 seeds were sown in case of 100% germination, and more seeds in case of lower germination. After sowing, a synthetic fertilizer was applied, providing 12:24:60 units NPK. During the establishment phase, weeds were controlled manually by hoeing and grass weeds were controlled with a single application of fluazifop-P-butyl (Fusilade Max^®^, Nufarm B.V., Belgium) at 1.5 lha^-1^. Due to dry weather in autumn 2018, germination was poor for some red clover accessions. Therefore, seedlings within poorly germinated plots were transplanted so that plants were equally distributed in the plot.

**Figure 2:**
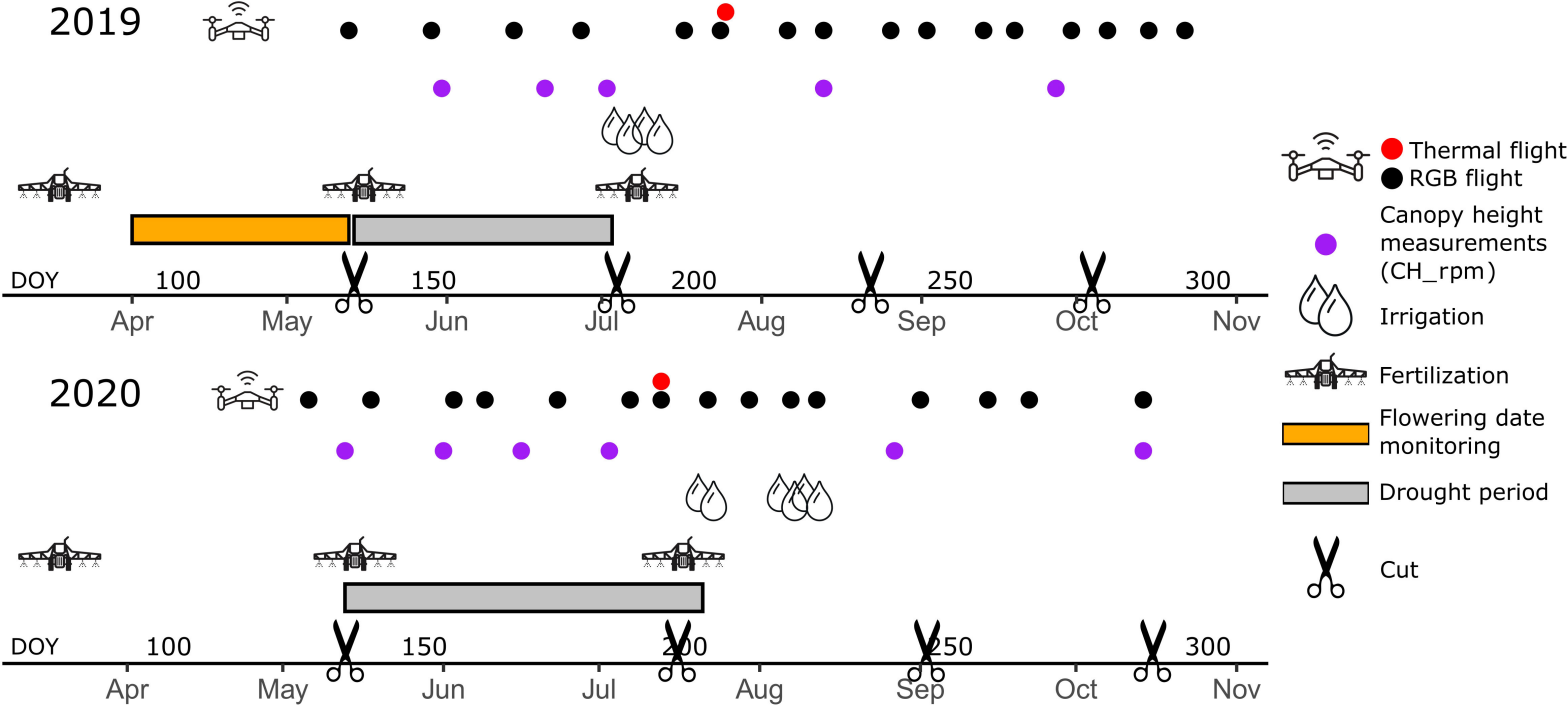
Timeline representing the two trial years for the drought and the control fields with schematic indication of the drought periods, mowing events, management, irrigation, and observations that were used in this study.

Agronomic activities, drought periods and observations are presented schematically in **Figure 2**. The crop was mown four times per growing season, as is usual practice in temperate European red clover. In the first production year (2019), further referred to as year 1, the crop was mown on DOY 134, 185, 232 and 277. In the second production year (2020), further named year 2, the crop was mown on DOY 134, 198, 246 and 290. Mowing was done with a Haldrup mower at 7 cm height. In year 1, weeds were controlled by carbetamide (Legurame^®^, Belchim Crop Protection, Belgium) at 5.0 lha^-1^ on DOY 51 and isoxaben (AZ500^®^, Dow Agrosciences, Belgium) at 0.1 lha^-1^ on DOY 78. Weed control was similar in year 2, with herbicide applications on DOY 69 and 106. Regular hoeing during the rest of the growing season kept the weeds under control. Both fields were fertilized in three doses with 0:45:328 units NPK on a yearly basis.

The drought treatment was initiated each year right after the first cut and maintained until soil moisture content reached 6% (v/v), close to the permanent wilting point (Loucks et al., 2018). At the end of the drought period, the second cut was taken and a modest irrigation was applied to the drought-treated field. In year 1, drought was maintained for 50 days from DOY 134 to 184. Due to unusual dry weather conditions, the control field was irrigated through sprinklers at a rate of 23 lm^-2^ on DOY 144, 152 and 167. After removal of the rain-out shelters, the drought field was irrigated through hosing at a rate of 10 lm^-2^ on DOY 185 and 191. In year 2, drought was initiated on DOY 134 and maintained for 69 days until DOY 203. The control field was irrigated with 23 lm^-2^ through sprinklers on DOY 143 and 152, except the first block that was accidentally provided 794 lm^-2^. This accidental irrigation did not seem to have greatly influenced our results: the effect was compensated by the block effects in our mixed model analysis. After removal of the rain-out shelters, the drought field was irrigated by sprinklers on DOY 204 (23 lm^-2^), 219 (29 lm^-2^) and 224 (32 lm^-2^).

**Figure 3:**
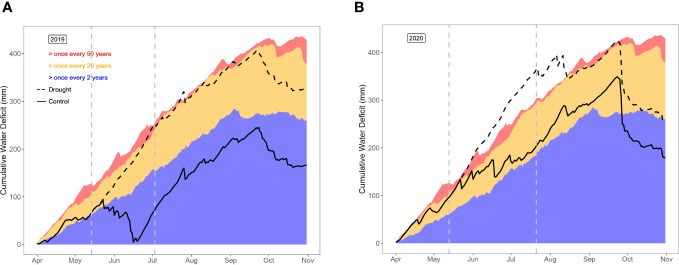
Cumulative water deficit in the control and drought fields in year 1 **(A)** and year 2 **(B)**, compared to historical data for the region of the trial.

Temperature, precipitation and daily solar shortwave radiation for the trial site were available from the Royal Meteorological Institute (KMI), in the form of interpolated data from nearby weather stations, the nearest station about 4 km away at 50°58’49”N, 3°48’57”E. The weather conditions in years 1 and 2 are summarized in **Supplementary Figure S1**. Precipitation and reference evapotranspiration data were used to calculate the cumulative water deficit (CWD) as described in Saleem et al. (2022) (**Figure 3**). To assess possible differences in soil water content between blocks, soil moisture content was monitored at regular occasions in the control and drought fields. At each event, 4 soil samples (0 – 30 cm) were taken per block, samples were oven-dried (70°C for 48h), and soil moisture was expressed as % (v/v).

### Data acquisition and processing

Ground and UAV-derived measurements were made in both fields throughout the entire duration of the trial. In year 1, visual observations were made for flowering date until the first cut (**Table 1**, **Figure 2**). Throughout the growing seasons of years 1 and 2, canopy height (CH_rpm) was measured several times in a representative part of each plot using a rising plate meter (RPM, HerboMETRE, ARVALIS‐Institut du Végétal, France) consisting of a square rising plate of 0.09 m² (30 × 30 cm). Additionally, regular UAV flights were performed to estimate the Canopy Cover (CC), Canopy Height (CH) and canopy temperature of each plot in the control and drought fields (**Table 1**). A total of 45 UAV flights was performed using a drone (model DJI Matrice 600 Pro; DJI, China) with an RGB sensor (α6000, 35mm lens, Sony Corporation, Japan) and a multispectral sensor (Micasense RedEdge MX RED in year 1 and Micasense RedEdge MX RED + MX BLUE in year 2). The flight altitude was set to 25 m, at a speed of 2.3 ms^-1^ and the side and forward overlap was 80-80%. Images were corrected and adjusted for white balance and exposure in Lightroom v6.5 (Adobe Systems Incorporated, USA) using a grey reference card (18% reference grey, Novoflex, Präzisionstechnik GmbH, Germany). The software Agisoft Metashape Professional v1.5.5 (Agisoft LLC, Russia) was used to stitch the images adding nine ground control points (coordinates measured with an RTK GPS Stonex S10 GNSS, Stonex SRL, Italy) to obtain a georeferenced orthomosaic and digital elevation model for each date. To extract data from each plot, rectangular polygons were created in QGIS 3.18.2 (QGIS Geographic Information System, QGIS Development Team, Open-Source Geospatial Foundation) and only pixels which corresponded to vegetation were considered. CC and CH were derived as in Borra-Serrano et al. (2020). For CH, Q90 values were used. Multispectral data were processed, but were not used further.

**Table 1:** General information on the variables recorded through ground observations and UAV-derived measurements.

Name	Abbreviation	Acquired through	Description	Unit
Flowering date	FLD	Ground observation	Day of year (DOY) until the start of flowering in year 1. Non-flowering accessions are encoded 222.	DOY
Canopy cover	CC	UAV-derived	Canopy cover. Percentage of the polygon covered by the canopy.	%
Canopy height	CH	UAV-derived	Canopy height. Height of the canopy relative to the naked soil (Q90).	Cm
Canopy Height	CH_rpm	Ground measurement	Canopy height. Height of the canopy relative to the naked soil measured with a rising plate meter.	Cm
Crop water stress index	CWSI	UAV-derived	Relative transpiration rate at the time of measurement	%

In order to assess the value of thermal data (CWSI values) for phenotyping drought tolerance in red clover, canopy temperature data were obtained from two successful thermal flights using a Wiris second generation (Workswell, Czech Republic) sensor. The two sets of thermal images (DOY 206 in year 1 - weather conditions 36°C and 38% RH; DOY 195 in year 2 – weather conditions 21°C and 54% RH) were pre-processed in ThermoFormat (Workswell, Czech Republic) and stitched in the software Pix4D Mapper 4.5.6 (Pix4D S.A., Switzerland). The CWSI (Maes and Steppe, 2012) was calculated as described in De Swaef et al. (2021).

### Data analysis

Variables were named using the variable abbreviation (**Table 1**) followed by the DOY (e.g. CC_178 for canopy cover measured on DOY 178). Both, ground measured and UAV-derived measurements were filtered for faulty plots for each variable, i.e. plots in which the crop was not completely established by the start of the growing season in year 1, plots that experienced damage during an exceptional herbivore attack, and plots with outliers. Data from a limited number of UAV flights was not reliable: these data were not used. In total, we decided to use data from 31 flights. The filtered phenotypic data was further harnessed for statistical analysis. The number of retained plots per variable is represented in **Supplementary Table S2**.

All statistical analyses were carried out in R statistical software version 4.0.3 (R Core Team, 2022), implemented in RStudio (RStudio Team, 2022). A mixed model approach with the lme4 package (Bates et al., 2015) was performed to correct for environmental effects caused by block and position within block (row and column), and to obtain best linear unbiased predictor (BLUP) values for each accession.

The following base model was considered: Y = Intercept + Accession + Block + Column + Row + Residual.

Where, ‘Y’ = response variable, ‘Intercept’ = overall mean value of the response variable, ‘Accession’ = random effect representing the accession, ‘Block’, ‘Column’, ‘Row’ = random effects representing spatial components in the experimental design and ‘Residual’ = noise term. The random effects for Block, Column and Row were assumed to be independent, and originate from an identical, normal distribution. The residuals are also assumed to be independent and identically distributed. In the experimental design, column was nested in block. The base model was not applied as such because it would be ‘overfitted’ (incorporates the ‘Block’ and ‘Column’ as unique components while in our design columns were actually nested). For each response variable, six versions of the base model were tested:

1. Y = Intercept + Accession + Residual
2. Y = Intercept + Accession + Block + Residual
3. Y = Intercept + Accession + Column + Residual
4. Y = Intercept + Accession + Row + Residual
5. Y = Intercept + Accession + Block + Row + Residual
6. Y = Intercept + Accession + Column + Row + Residual

For each variable, the output was evaluated using the Akaike Information Criterion (Akaike, 1974), and the fit with the lowest value was chosen. From the best fit, the BLUP values were calculated for each accession as the sum of the ‘Intercept’ value and the value of the random effect of ‘Accession’. The variance components of the fit were obtained using the VarCorr function from lme4 package (Bates et al., 2015). The broad sense heritability (H²) was calculated from the variance components of the best fit as follows: H^2^ = V_G_/(V_G_ + V_R_), where V_G_ and V_R_ represent the variance due to ‘Accession’ and the residual variance, respectively. For each variable, the model used and the heritability is given in **Supplementary Table S2**.

### Relative performance index Yr

Drought responses were measured in terms of growth reduction (using the proxies CC and CH) compared to control conditions (Bellague et al., 2016). To assess plant responses to drought, relative performance indices (Yr) were calculated for the UAV-derived observations CC, CH and CWSI, using the following formula: Yr = (Control – Drought)/Control. Yr variable names are composed of ‘Yr’ followed by the variable (CC, CH or CWSI) and the DOY (e.g. Yr_CC_178 for relative performance indices for canopy cover measured on DOY 178). A positive Yr value for a certain accession indicates a lower observation in terms of CC, CH or CWSI for that accession in the drought field compared to the control field, and vice versa. Two-sided Z-tests were used to assess for each variable and observation date whether average Yr values over all accessions differed statistically from zero, i.e. indicating no growth reduction due to drought.

### Principal component analyses

Principal component analysis (PCA) was used to understand the relationships among variables in this complex dataset. First, PCA was performed on the data of the control field for each year separately to understand patterns of clustering and relations among CC, CH, maturity, variety type and geographic origin. At first, we included all observations for CC and CH between the 1^st^ cut (start of drought) and the 4^th^ cut (end of the growing season). As CC and CH observations taken during the same growing period were generally well correlated, we reduced the number of variables to 1 observation in the middle and 1 observation at the end of each of the three growing periods. By doing so, we obtained a set of informative CC and CH variables that explained an equal proportion of variation in the PCAs as the complete set of variables. In year 1, we retained observations for CC and CH on DOY 165, 178, 205 225, 256, and 273. In year 2, we retained observations for CC and CH on DOY 161, 195, 220, 245, 266, and 288.

Second, PCA was performed on the dataset of Yr indices to reveal key variables and time-points related to drought responses, using our informative set of CC and CH variables describes above, and the CWSI variables. FactoMineR (Lê et al., 2008) and factoextra (Kassambara and Mundt, 2020) packages in R were used to depict patterns related to maturity (early – late), variety type (breeding material, cultivars, landraces or ecotypes), and geographic origin (N Europe, W Europe, Central Europe, SE Europe, or Non-European material).

### Relation between growth patterns under control and drought conditions

An important question is whether breeders can create varieties with improved performance under drought conditions by breeding in control conditions. To investigate this, we ranked all accessions according to the number of CC and CH variables for which they attained a value in the top 25%. In other words, as we had determined CC and CH at different moments in the two growing seasons, the number of times each accession ranked among the top 25% best-performing was counted. This allowed to identify the accessions with best overall performance for CC or CH in the control and drought fields in both years: accessions which a breeder would identify as ‘promising’. For both years and both fields, we identified the 50 accessions (or slightly more in case of accessions with equal number of hits in the top 25%) with best overall performance in terms of CC and CH. This allowed to assess how many accessions in this subset performed best in both fields.

## Results

### Weather conditions and drought intensity in both trial years

Daily average temperatures and daily solar shortwave radiation for year 1 and year 2 are presented in **Supplementary Figure S1**. Year 1 (2019) and year 2 (2020) were warm, sunny and relatively dry with a warm, early spring and a warm summer. Daily average temperatures exceeded the long-term average of 10.6°C by 0.9°C and 1.6°C on a yearly basis, and total yearly precipitation was 54 mm and 121 mm below the long-term average of 852 mm, for year 1 and 2, respectively.

The weather conditions before, during, and after the drought periods were similar in both years. Before the drought treatment (DOY 1 to 134), both years displayed similar daily average temperatures (7.6 vs. 8.4°C), daily solar radiation (9.2 vs. 10.1 MJm^-2^) and precipitation (248 vs. 280 mm), for year 1 and 2, respectively. During the drought periods (DOY 134-184 and 134-203 in year 1 and 2, respectively), daily average temperatures (16.5 vs. 16.6°C), daily solar radiation (20.4 vs. 20.7 MJm^-2^) and cumulative precipitation (112 vs. 114 mm) were also similar. Both drought periods contained 12 warm days with maximal temperatures exceeding 25°C, and respectively 4 and 1 hot days with maximal temperatures exceeding 30°C. Major rainfall events during the drought periods occurred on DOY 156 (15.1 mm) and 165 (27.6 mm) in year 1, and DOY 169 (36.8 mm) and 182 (16.0 mm) in year 2. The recovery phases after the drought treatments and the remainder of the growing seasons (drought relief to DOY 300) remained warm in both years, with similar daily average temperatures (16.7°C in both years), daily solar radiation (13.4 vs. 12.5 MJm^-2^) and precipitation (222 vs. 257 mm), for year 1 and 2, respectively. Multiple hot days with daily maximum temperatures exceeding 30°C occurred during the recovery phases: 8 days in year 1 and 9 days in year 2.

The cumulative water deficit (CWD) represents the soil moisture stress, by taking into account evapotranspiration, precipitation, irrigations applied in the control field, and the effect of the rain-out shelters in the drought field (**Figure 3**). In year 1, the control field represented nearly normal soil moisture conditions, with a short and modest CWD in early summer. The drought field, on the other hand, suffered a once-in-20-year-drought starting soon after the installation of the rain-put shelters until the end of the growing season, long after removal of the rain-out shelters. In year 2, drought was more harsh in both fields. In the control field, the CWD entered the once-in-20-year-drought zone early in the growing season, and remained at the margin of that zone until late in the growing season. The drought field suffered even more soil moisture stress, representing a once-in-50-year-drought from the installation of the rain-out shelters until mid-August. In other words, year 1 represents a comparison between normal (control field) vs. dry conditions (drought field), whereas year 2 compares dry (control field) vs. severely dry soil moisture conditions (drought field).

The actual soil moisture content was monitored during the drought treatments. In year 1, soil moisture in the drought field dropped from 11% to 6% (v/v) during the drought period. In year 2, soil moisture levels were already lower at the start of the drought period, and dropped from 8% to 7% (v/v). In year 2, around three weeks after drought relief (DOY 224), soil water contents were still low in both fields: 16% in the control field vs. 11% in the drought field (v/v).

### Correlations between ground-measured and UAV-derived canopy height data

The rising plate meter is considered a reference method to measure plant height in phenotyping studies. UAV-derived measurements could produce the same data in considerably less time, but this method has not been thoroughly studied or validated in red clover. Therefore, we evaluated whether it was possible measure canopy height through UAV-derived measurements in red clover. Pearson correlation coefficients between ground-measured (CH_rpm) and UAV-derived (CH) measurements for canopy height were high in both years and in both fields (average over the different time points r = 0.89, lowest value r = 0.80), even for observations taken up to 7 days apart (**Supplementary Table S3**). Therefore, we decided to continue only with the UAV-derived CH measurements, as they included many more observations.

### Performance under control conditions

**Figure 4:**
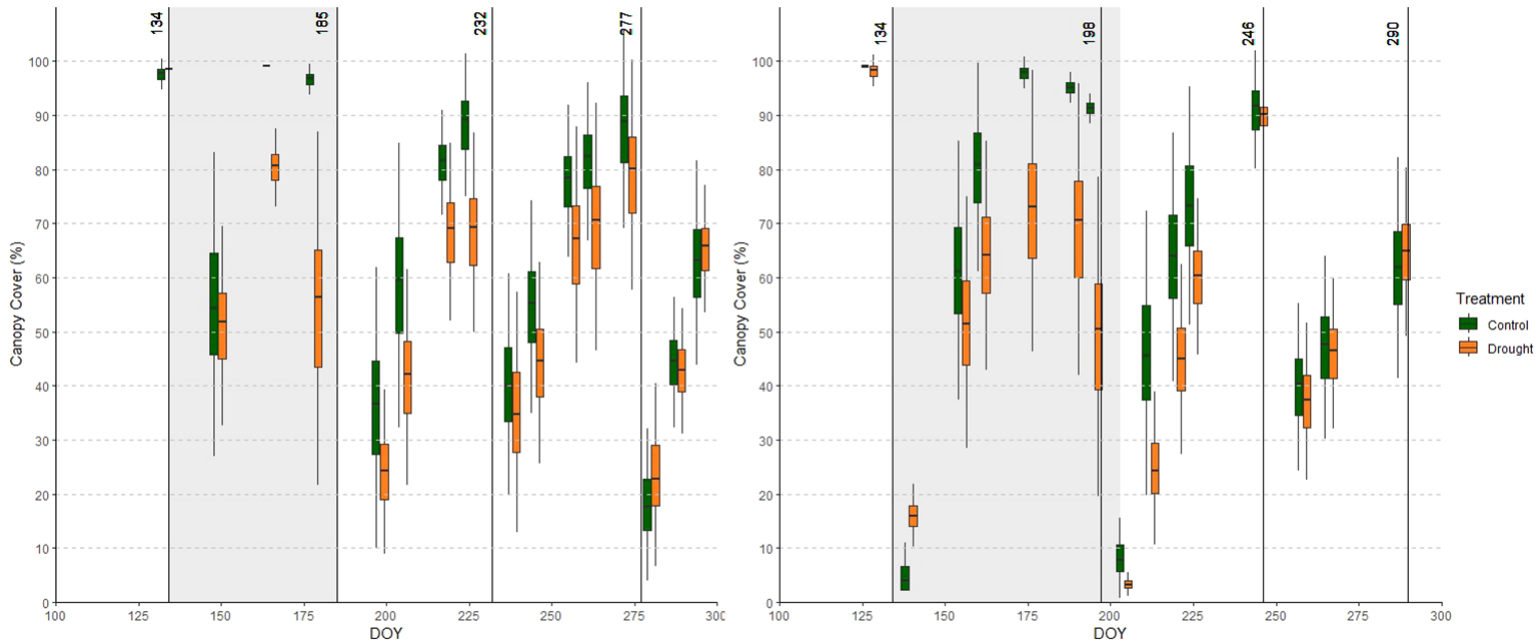
Average canopy cover (CC) over all accessions in the relevant part of the growing season of year 1 (left) and year 2 (right) in the control (green) and drought (orange) fields. Adjacent green and orange boxplots represent measurements in the control and drought fields obtained on the same day. Cuts are indicated with vertical lines and drought periods are shaded.

**Figure 5:**
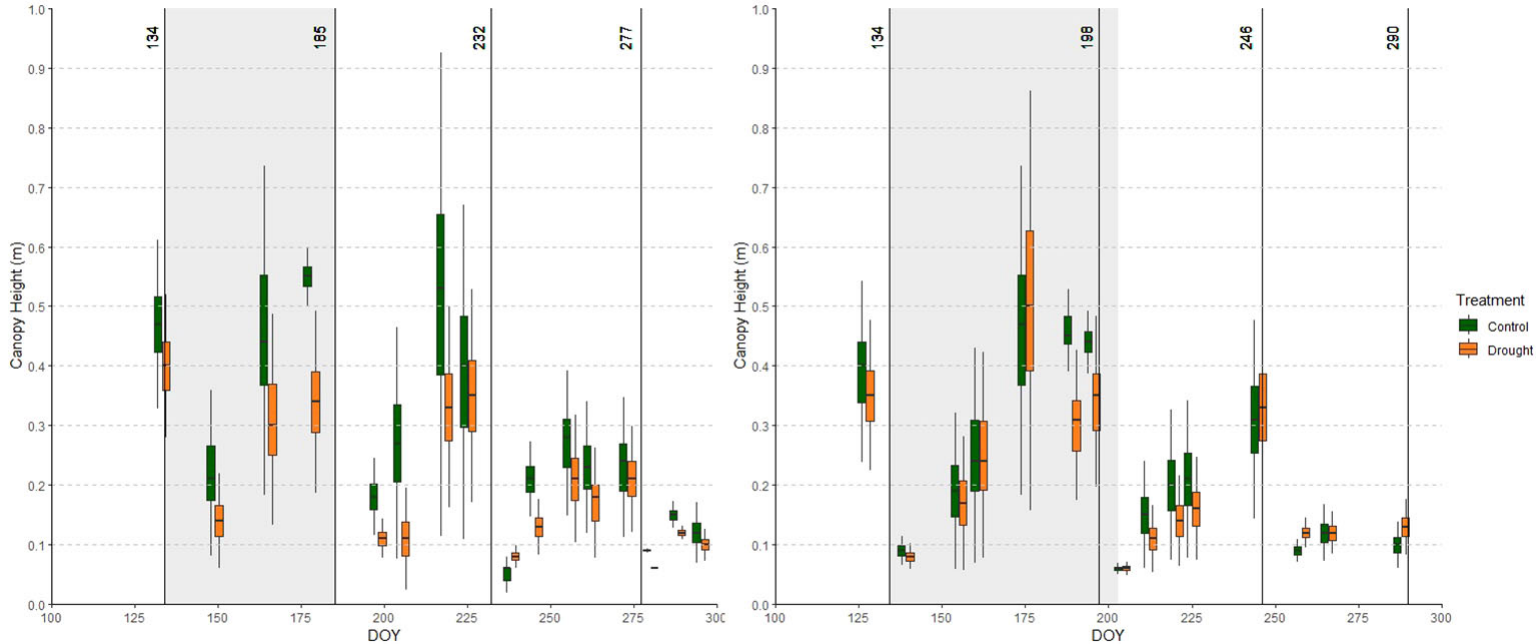
Average canopy height (CH) over all accessions in the relevant part of the growing season in year 1 (left) and year 2 (right) in the relevant part of the growing the control (green) and drought (orange) fields. Adjacent green and orange boxplots indicate measurements in the control and drought fields obtained on the same day. Cuts are indicated with vertical lines and drought treatments are shaded.

Before analyzing the response of this red clover panel to drought, we investigated the general performance of the accessions in the control field. CC and CH were low during the winter and at the start of the growing season. During the growing season, CC increased to nearly 100% before each cut (**Figure 4**). Similarly, CH increased during each growing period, and reached a plateau towards the end of each growing period. In some cases a decline in CH was observed shortly before mowing (e.g. cut 1 in year 1), which can be attributed to the bending (or lodging) of the sward (**Figure 5**). Maximal canopy height reached 75 cm in some accessions. For CC, and CH to a lesser extent, a high degree of variability among accessions was present in the beginning of each growing period, which shrunk towards the end of the growing period. This implies that some accessions quickly re-grew after mowing, and quickly recovered to maximum CC (and CH), whereas other accessions needed more time to restore their maximum CC and CH. Broad-sense heritability values were always low just after mowing, increased during the growing period, and were maximal right before the next cut (**Supplementary Table S2**). This indicates that measurements at the end of each growing period represent best the genetic variation present in this collection. Growth patterns differed little between year 1 and 2. CC before the 4^th^ cut was lower in year 2 than in year 1 (62% vs. 88% for CC_288 in year 2 and CC_273 in year 1, respectively). On average, CH before each cut was lower by approximately 10 cm in year 2.

### Drought stress responses

Accession averages for all observations under control and drought conditions, together with the Yr indices are given in the **Supplementary Materials**. In both years, drought substantially inhibited plant growth, which became observable already two weeks after the initiation of the treatment. Drought-treated plots produced a less dense and shorter canopy than control plots. Leaf wilting was only observed by the end of the drought treatment, and only to a limited extent (personal observation). Drought effects were especially evident in year 1.

Drought caused reductions in CC: 19% and 44% at CC_165 and CC_178 in year 1, and 26% and 46% at CC_175 and CC_195 in year 2, respectively (**Figure 4**). In addition, we observed a negative effect of drought on CC in the growing period following the treatment (between the 2^nd^ and 3^rd^ cut) in both years. In year 1, drought also tended to affect CC in the growing period between the 3^rd^ and 4^th^ cut, yet this effect had disappeared in year 2. The Yr indices for CC (**Table 2**) confirm this trend: in both years, the relative performance of the drought field gradually decreased as the drought period progressed, and remained lower during the recovery phase.

**Table 2:** Relative performance indices (Yr) for CC, CH and CWSI, averaged over all accessions, for different observation moments in the growing seasons of year 1 and year 2.

Year	DOY	Average Yr index			
		Yr_CC	Yr_CH	Yr_CWSI	
2019	133	-0.009^***^	0.148^***^		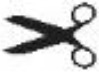
2019	149	0.057^***^	0.351^***^		
2019	165	0.178^***^	0.311^***^	
2019	178	0.438^***^	0.373^***^		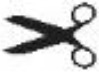
2019	198	0.303^***^	0.351^***^		
2019	205	0.280^***^	0.593^***^	
2019	206			-0.735^***^
2019	218	0.155^***^	0.349^***^	
2019	225	0.221^***^	0.069^***^		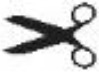
2019	238	0.123^***^	-0.586^***^		
2019	245	0.185^***^	0.394^***^	
2019	256	0.146^***^	0.231^***^	
2019	262	0.144^***^	0.227^***^	
2019	273	0.092^***^	0.106^***^		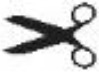
2019	280	-0.328^***^	0.289^***^		
2019	288	0.031^***^	0.186^***^	
2019	295	-0.046^***^	0.095^***^	
2020	127	0.008^***^	0.114^***^		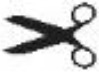
2020	155	0.154^***^	0.061^***^		
2020	161	0.202^***^	-0.007	
2020	175	0.258^***^	-2.750^***^	
2020	189	0.275^***^	0.332^***^	
2020	194			-1.280^***^
2020	195	0.462^***^	0.225^***^		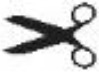
2020	204	0.568^***^	0.004		
2020	212	0.454^***^	0.269^***^	
2020	220	0.291^***^	0.305^***^	
2020	225	0.174^***^	0.206^***^	
2020	245	0.013^***^	-0.043^***^		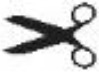
2020	258	0.059^***^	-2.750^***^		
2020	266	0.017^**^	-0.005		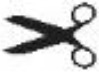
2020	288	-0.054^***^	-0.301^***^		

DOY, day-of-year; CC, canopy cover; CH, canopy height; CWSI, crop water stress index; Yr, relative performance index; SD, standard deviation. Mowing events are indicated with dashed lines and scissors. Drought periods are shaded. Asterisks indicate if Yr values differ statistically from 0 according to Z-test: ^*^: p < 0.05; ^**^: p < 0.01; ^***^: p < 0.001.

Asterisk indicate if Yr values differ significantly from 0 according to Z-tests.

The effects of drought on CH are shown in **Figure 5**. Before the drought treatment in year 1, the control field slightly outperformed the drought field in terms of CH. This is also represented in the Yr index (**Table 2**), which was higher than 0 at the start of the season in year 1. Nonetheless, drought caused clear reductions in CH, especially in year 1: 32% and 37% at CH_165 and CH_178 in year 1, and 9% and 9% at CH_175 and CH_195 in year 2. Similar to CC, a legacy effect of drought was observed in the two growing periods following the drought in year 1 (less mature swards), but only in the first growing period after drought in year 2 (more mature swards). As the stress intensity in the two years was different, it is difficult to separate the effects of sward age and stress intensity.

In both years, the Yr indices for CH increased towards the end of the drought period. Similar to CC, a legacy effect was observed in the drought field: CH in the drought field remained substantially lower than in the control field until the 4^th^ cut.

Whereas the CC data for each time point generally approached a normal distribution, CH displayed a bimodal distribution in the control field in the growing periods between cuts 1 and 2, and cuts 2 and 3, i.e. in late spring and summer. More precisely: in year 1 at CH_149, CH_165, CH_205, CH_218 and CH_225, and in year 2 at CH_139, CH_155, CH_161, CH_175, CH_212, CH_220, CH_225, and CH_245 (**Supplementary Figure S2**). This segregation between rapidly and slowly re-growing accessions was investigated further in our PCA.

Due to technical reasons, less thermal UAV flights could be performed than initially intended. We obtained CWSI data only on two occasions. In year 1 CWSI data were obtained 22 days after drought relief (CWSI_206), and in year 2 at the end of the drought period (CWSI_194). Average CWSI values for the control and drought field were 0.39 vs. 0.67 (CWSI_206 in year 1), and 0.16 vs. 0.37 (CWSI_194 in year 2). This shows that on the days these measurements were done there was a clear difference between control and drought fields in both years. In other words, at both occasions stress levels experienced by the plants were higher in the drought field compared to the control field.

### Relations between variables

**Figure 6:**
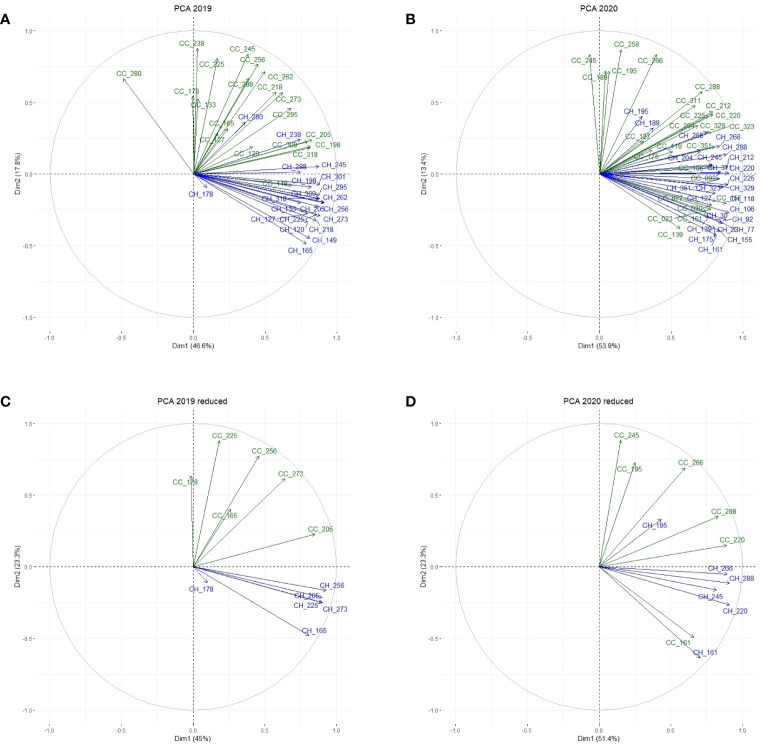
PCA on the control field displaying the relations between all observations for CC (green) and CH (blue) between the first and last cut in year 1 **(A)** and year 2 **(B)**, and between observations in the middle and at the end of each growing period between the 1^st^ and 4^th^ cut in year 1 **(C)** and year 2 **(D)**.

General relations among CC and CH variables were uncovered by our PCAs on the control field data. In the first step, PCAs were performed including all observations for CC and CH between the 1^st^ and the 4^th^ cut (i.e. the drought period and the recovery phase) (**Figures 6A, B**). The first two principal components explained 64.4% and 67.2% of the total variation in year 1 and year 2, respectively. Subsequently, the number of variables was reduced to 1 representative observation in the middle and 1 at the end of each growing period (**Figures 6C, D**). In the latter set of PCAs, the first two principal components explained 68.3% and 74.7% of the variation, respectively in year 1 and year 2. We decided to continue with the reduced set of PCAs, as this set contained sufficient variables to describe the growth responses in our panel of accessions. Generally, the relations between variables in the control field were similar, but not identical in both years. In year 1, CC measurements at different dates were correlated among themselves, and largely independent of CH measurements. In year 2, the separation between CC and CH was less distinct than in year 1 (e.g., CC_161 and CH_161 were highly correlated).

**Figure 7:**
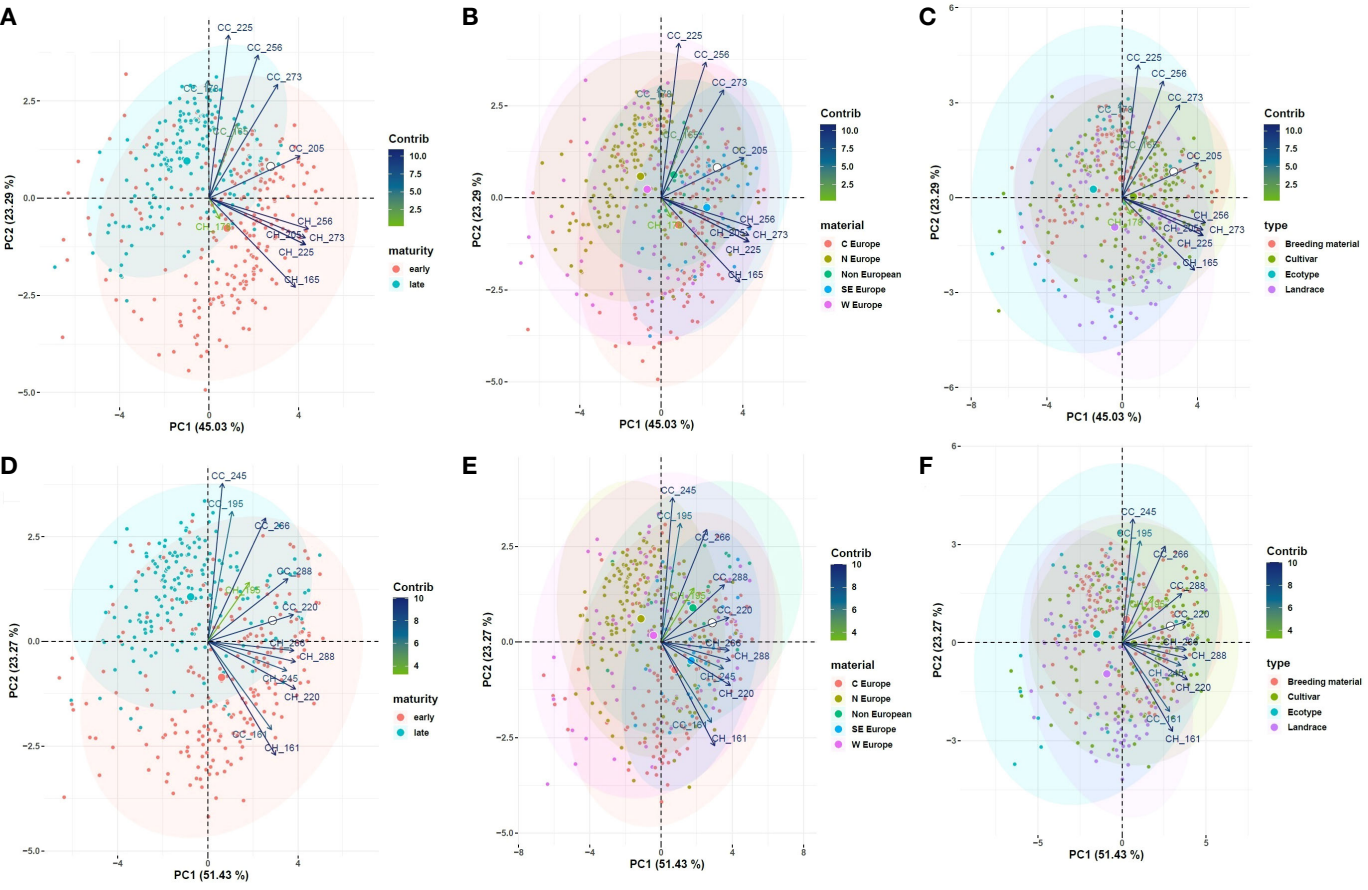
PCA on the control field for relevant observations of CC and CH, displaying the clustering according to maturity **(A)**, geographic origin **(B)** and variety type **(C)** in year 1 and in year 2 (**D–F**, respectively). Contrib: contribution (%) of each variable to the principal components.

Clustering according to maturity, geographic origin, and variety type is represented in **Figure 7**. A clear clustering according to maturity was found: in both years, early flowering accessions generally attained higher CH, while late flowering accessions had higher CC values. This is likely due to the formation of flowering stems on relatively small plants in early accessions, while late accessions produced more leafy branches before initiating flowering. Clustering patterns for maturity and geographic origin corresponded well: as expected, accessions from Central and South Europe were mostly early flowering, accessions from North Europe were mostly late flowering, and most West European and non-European accessions were situated in-between. No clear clustering was found according to variety type (ecotype, landrace, cultivar or breeding material), which indicates that relations among the investigated variables are similar in all variety types.

**Figure 8:**
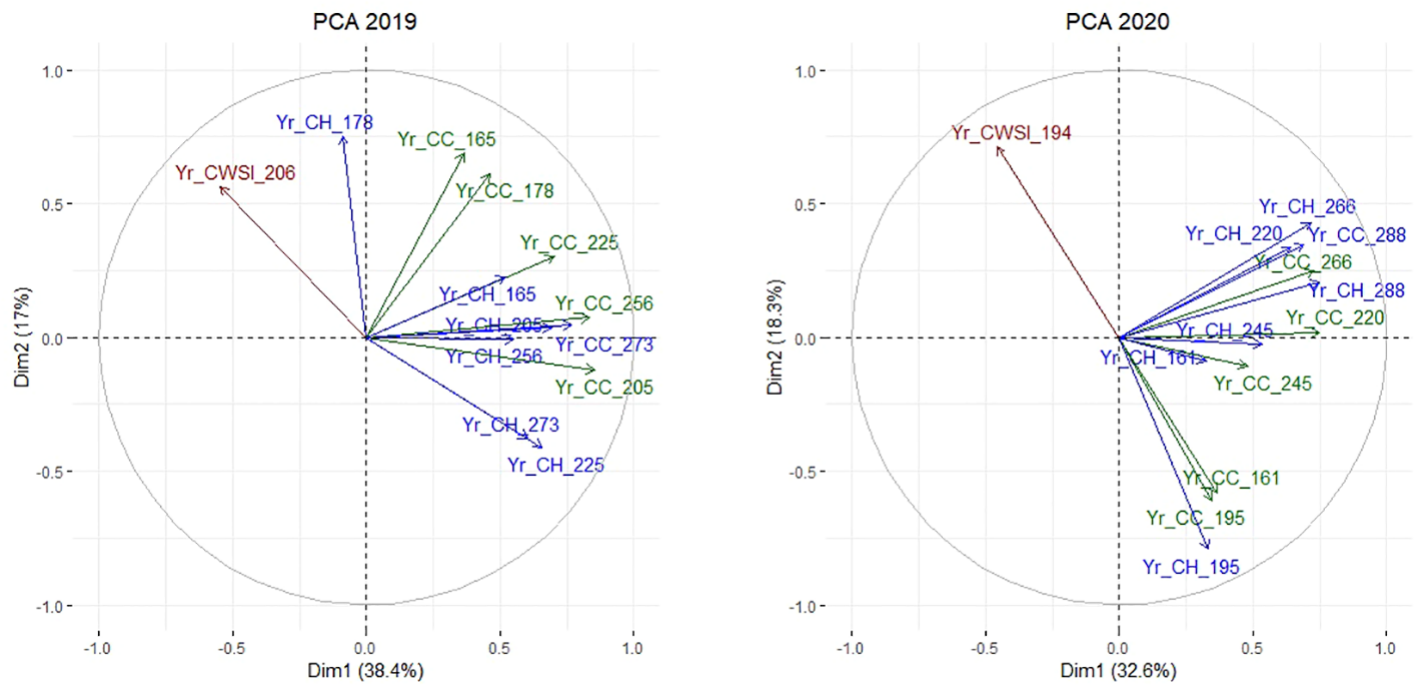
PCA on the Yr indices in year 1 (left) and year 2 (right) displaying relations between CC (green) and CH (blue) in the middle and at the end of each growing period, and CWSI (red).

A final set of PCAs was performed on the Yr indices to reveal key variables and time-points related to drought responses. The general patterns of differentiation between Yr_CC, Yr_CH, and Yr_CWSI were similar in year 1 and year 2 (**Figure 8**), indicating similar responses to drought stress in both trial years. In both years, Yr_CC and Yr_CH observations clustered into two rather independent groups: observations during drought (Yr_CC and Yr_CH observations on DOY 165 and 178 in year 1, and on DOY 161 and 195 in year 2), and during the recovery phase after drought (Yr_CC and Yr_CH observations on DOY 205, 225, 256 and 273 in year 1, and on DOY 220, 245, 266 and 288 in year 2). In other words, plant responses during the drought period and in the recovery phase were largely independent. In general, CC and CH observations acquired on the same day were fairly well correlated in both years (e.g., Yr_CC_256 and Yr_CH_256 in year 1), except in late spring and early summer (e.g. Yr_CC and Yr_CH on DOY 165 and 178 in year 1), probably due to the effect of flowering which causes an increase in CH but not necessarily in CC. Hence, Yr_CC and Yr_CH could be used interchangeably to phenotype drought responses in late summer and autumn. In both years, Yr_CWSI observations were inversely correlated to CC and CH measurements acquired in the same period. (e.g., CWSI_206 vs. Yr_CC_205, Yr_CH_225 in year 1, and Yr_CWSI_194 vs. Yr_CC_195, Yr_CH_195 and Yr_CC_161 in year 2).

### Relation between growth patterns under control and drought conditions

When we ranked the accessions according to the number of observations for CC or CH for which they attained value in the top 25%, it became possible to identify the 50 (or slightly more in case of accessions with equal performance) accessions with best overall performance, i.e. the ‘top’ accessions, in the control and drought fields (**Table 3**). Subsequently, we assessed how large the overlap was between both groups, i.e. how many accessions ranked in the top groups of both fields. Generally, nearly half of the accessions from the top group of the drought field also ranked top the control field, and vice versa. For CC, the percentage of top accessions in the control field that ranked top in the drought field was lower in year 1 compared to year 2 (18/60 = 35% vs. 27/52 = 46%, respectively). For CH, the overlap was similar in both years (25/52 = 46% in year 1 vs. 27/50 = 54% in year 2).

**Table 3:** Accessions with overall best performance in terms of CC and CH in the control field, in the drought field, and in both fields for both years.

Year	Variable	# Accessions classified as top ranking in		
		Control field	Drought field	Both fields
2019	CC	60	51	18
2020	CC	52	59	27
2019	CH	52	54	25
2020	CH	50	50	27

## Discussion

### Meteorological conditions during the trial

Years 1 and 2 (2019 and 2020) were classified by the KMI (Royal Meteorological Institute, Belgium) as ‘relatively dry’ and ‘dry’ (KMI, 2020; KMI, 2021). Year 2 inhibited higher average temperatures and less precipitation, and imposed more soil moisture stress than year 1. In contrast to what we intended, the control field in year 2 also suffered above-normal soil moisture stress, in spite of the (modest) irrigations applied. In retrospect, we should have irrigated the control field more in year 2 to simulate normal soil moisture conditions. Nonetheless, the drought field suffered even more soil moisture stress, rendering Yr observations for year 2 meaningful.

### Performance under control conditions

The control field showed similar patterns of CC and CH during both growing seasons (**Figures 4**, **5**). However, in year 2 CC was lower before the 4^th^ cut, and CH before each cut remained lower by approximately 10 cm compared to year 1. These differences are presumably due to the (unintended) soil moisture stress in the control field in year 2, which appear to have impacted plant growth. Furthermore, we observed that CC and CH behaved more independently from each other in year 1 compared to year 2 (**Figure 6**). This could also be due to the drier conditions in year 2, or it may be explained by the fact that the perennial plants in our plots were older in year 2 than in year 1.

### How to phenotype red clover drought responses?

Implementation of High-Throughput Field Phenotyping (HTFP) using a UAV-based phenotyping protocol using RGB and thermal sensors was extremely advantageous in a study where plants need to be phenotyped in a dynamic way over the course of an entire growing season, as in the present work. Getting a similar dataset using destructive measurements of biomass yield or manual measurements of CC or CH would have been extremely time-consuming. Although we did not validate the CC observations using an destructive method (cutting and weighing), we have shown that UAV-derived CH observations were highly correlated with manual measurements (CH_rpm) using a rising plate meter. Similar findings were obtained in forage grasses by Borra-Serrano et al. (2019). This suggests that our UAV protocol to measure CH can be applied in a reliable way in red clover.

All variables studied – CC, CH and CWSI – were clearly affected by drought. Reductions in CC and CH presented themselves two weeks after the onset of the drought treatment, and remained observable until the 4^th^ cut in year 1, and until the 3^rd^ cut in year 2. The PCA revealed that CC and CH were largely independent variables that may describe different genetic variation.

During the drought period, relative reductions in CH were smaller than for CC. A first explanation is that CH at the end of a growth period can be affected by lodging, which can ‘conceal’ actual differences between control and drought-treated plants. Presumable lodging effects were observed in the control field in year 1 before the 2^nd^ (CH_178) and 3^rd^ cut (CH_225), and in year 2 before the 2^nd^ cut (CH_195). In the drought field, on the contrary, no lodging was observed and CH continued to slowly increase towards the end of each growth period. Secondly, flowering can interfere with CH measurements. Numerous accessions flowered towards the end of the drought periods. In the control field, plants formed multiple flowering stems that were branched and rich in leaves and flower heads (personal observation – data not shown). In contrast, drought-treated plants generally formed fewer, more slender, but often reasonably high flowering stems. When measuring CH manually, it is possible to ‘straighten’ plants and bypass the effects of lodging and/or flowering. Although it is possible to flatten/smooth UAV-derived CH data and omit single high stems, our data displayed moderate reductions in CH in the drought-treated plants, while in fact the canopy density was remarkably lower than in the control field.

In addition, we have shown that measurements of canopy temperature using a thermal sensor mounted on a drone are very useful to determine the response of red clover plots to drought. A prerequisite is that thermal images are obtained on a warm and sunny day around solar noon, which is not always straightforward (Maes and Steppe, 2012). Due to technical failures and practical limitations, we could only obtain reliable canopy temperature data from two UAV flights. Nonetheless, CWSI values are a highly relevant variable to phenotype drought responses: as they reflect transpiration rates, they present a complementary source of information for selection. In year 1, CWSI data obtained after drought relief indicated that the drought-treated plants had not yet recovered from drought stress at that time and that their physiological performance was yet affected by the drought treatment. In year 2, CWSI data obtained at the end of the drought period indicated clearly higher stress levels in drought-treated vs/control plots. Both observations are in agreement with our observations for CC and CH. Congruently, previous work in forage grasses also found good correlations between CWSI values and breeders scores for plant vigor at the end of the drought period (De Swaef et al., 2021).

In conclusion, CC and CWSI are highly suitable variables to phenotype drought responses in red clover. Although CH is prone to bias, it can be used after correction for lodging and/or flowering. The most suitable time points to assess the response to drought is towards the end of the drought period, and/or during the recovery phase 2 to 3 weeks after drought relief, as reductions in CC and CH were maximal in these periods. Breeders aiming to phenotype drought responses in red clover can reduce time and resources by limiting the number of observations to these time points.

### Do we need drought trials to identify drought-tolerant plants?

When studying the ‘top’ group of accessions in the drought field, roughly half of them also ranked in the top group in the control field. This was true for CC and CH, but more pronounced for CH. In year 1, this is a remarkably finding, given the contrasting soil moisture stress levels in the control and drought fields. In year 2, a better relation can be expected, as the control field also imposed soil moisture stress, although less intense than the drought field. This finding implies that, when breeders select in normal soil moisture conditions, roughly half of the material that they select will also perform well under drought stress. In other words, even without applying drought conditions, drought responses will improve (to a certain extent), merely by selecting well-performing plant material.

### Associations between drought responses and accession characteristics

When a diverse panel of accessions is compared for a stress factor in a field trial, it is difficult to separate the stress response from confounding factors such as the accessions’ general adaptation, the variety type or the maturity type. A first factor that may interfere with stress responses is the accessions’ general adaptation, i.e. adaptation to the climate, soil type, mowing regime, local disease pressure, and other factors. Poorly adapted accessions may have suffered more background stress in our trial, which could interfere with their drought responses. However, our PCAs did not clearly show different responses in terms of CC and CH in material from regions different than West Europe (**Figure 7**).

A second factor is the variety type: ecotypes, landraces, cultivars and breeding material. Our PCA revealed no clustering of Yr indices according to the variety type, suggesting a similar response to drought in all groups. Ecotypes generally have poorly productive, short, and early-flowering phenotypes, while landraces, cultivars and breeding material develop taller, bushier and more productive plants (Taylor and Quesenberry, 1996). For breeders aiming to improve adaptation to drought in red clover, it is important not only to observe relative reductions in productivity after drought treatment, but also to monitor the absolute productivity of their plant material. Annicchiarico and Pagnotta (2012) compared various Italian red clover accessions for drought responses in a Mediterranean climate. Natural populations that evolved in regions with severe summer drought showed increased yield and persistence, but had no yield disadvantage relative to the best-performing landrace or cultivar.

A third interfering factor is the maturity type, which determines the capacity to re-grow after mowing. We observed bimodal distributions in CH in the control field between cuts 1 and 2 in late spring, and between cuts 2 and 3 in summer (**Supplementary Figure S2**), which coincided with the accessions’ maturity. After mowing, the rapidly re-growing and early flowering ‘double-cut types’ quickly formed flowering stems, whereas the ‘single-cut types’, most of them of Nordic origin, displayed slower regrowth and later flowering, with lower CH values as a consequence. This bimodal pattern was less apparent in the drought field, probably due to the effects of drought. Our PCA revealed that Yr_CC and Yr_CH observations at the end of the drought period acted largely independently from observations during the recovery phase. Simply put, accessions that reduced their growth during the drought period, did not necessarily reduce growth during the recovery phase, and vice versa. Previous studies in red clover have proposed two strategies: ‘drought tolerance’ and ‘drought survival’, which seem to coincide largely with the maturity type. Loucks et al. (2018) observed that double-cut red clover plants maintained growth longer during drought, but showed earlier and higher mortality than single-cut plants. In other words, double-cut types express more drought tolerance, while single-cut types focus more on survival (Loucks et al., 2018). As our PCA indicated, both strategies appear to be largely independent mechanisms in red clover, as proposed by Aslam et al. (2015). A similar pattern exists in alfalfa. Non autumn-dormant alfalfa accessions exhibit high drought tolerance, as they maintain productivity during drought, but display more mortality and slower recovery after severe drought stress (Pembleton and Sathish, 2014). Autumn-dormant alfalfa accessions show high drought recovery: they cease growing when drought sets in, but display low mortality and a quick re-growth after drought relief (Pembleton and Sathish, 2014). While under well-watered conditions non-dormant types often outperform dormant types, the opposite occurs under moderate to severe drought stress (Pembleton and Sathish, 2014). Our results also confirm the independent strategies and their possible association with the maturity type in red clover. However, it is difficult to make statements on plant mortality, as the number of plants per plot was not monitored in our trial.

### Consequences of a two-year experiment

Drought was imposed to the perennial plants in two subsequent years, and year 2 exhibited a higher soil moisture stress (CWD) than year 1. As the stress intensity in the two years was different, it is difficult to separate the effects of sward age and stress intensity. On the one hand, the soil moisture stress in the control field in year 2 may have concealed differences between the control and drought treatments. This reasoning may explain (1) why reductions in CH were larger in year 1 compared to year 2 (37% and 9% at the end of the drought period, respectively), and (2) why the legacy effect of drought on canopy cover and canopy height appeared larger and lasted longer in year 1 compared to year 2. Alternatively, plants in year 2 were older, and have had more time to develop deep roots, enabling them to access deep soil water. In the drought field, the drought in year 1 may even have triggered plants to increase their rooting depth, which undoubtedly helped them through the drought period in year 2. Especially well-adapted accessions that withstood the drought stress in year 1 well, would have had this opportunity. Additionally, it is likely that some selection for drought adaptation has occurred in year 1: the most drought-sensitive plants may have perished, allowing more drought-tolerant neighboring plants in the same plot to fill the gaps. This may explain why even the ‘objective’ index of drought stress (Yr_CWSI) was less pronounced in year 2 than in year 1.

## Conclusions and future perspectives

In the present paper, we characterized the responses to drought stress in a diverse panel of red clover accessions, and we identified promising accessions that could be used as source for breeding. We validated the use of UAV-derived measurements for CH in phenotyping drought responses. We observed largely similar responses to drought stress in the first and second production year, and we found evidence for two independent strategies to cope with drought stress, drought tolerance and drought recovery, which largely coincide with the maturity type. Finally, we pinpointed variables and time-points that are helpful to breeders aiming to create more drought-resilient red clover varieties. We further found that a large proportion of the accessions able to perform well under well-watered conditions were also the ones which were less affected by drought. However, it remains to be investigated which physiological mechanisms contribute to improved drought responses in red clover. Analyzing the multispectral data available for our trial could reveal additional characteristics such as the leaf density of the canopy, which could help to uncover physiological mechanisms behind drought responses. Furthermore, understanding the genetic basis of drought responses may provide additional insights and could reveal candidate genes associated with adaptation to drought stress. A next step could be the development of molecular markers, which would allow breeders to further optimize their breeding methods for adaptation to drought in red clover. In future research, we plan to perform GWAS for drought responses on the EUCLEG red clover collection, using the information generated in this study.

## Data availability statement

The original contributions presented in the study are included in the article/**Supplementary Material**. Further inquiries can be directed to the corresponding author.

## Author contributions

TV: Conceptualization, Data curation, Formal Analysis, Investigation, Methodology, Project administration, Supervision, Visualization, Writing – original draft, Writing – review & editing. AS: Conceptualization, Data curation, Formal Analysis, Investigation, Methodology, Software, Visualization, Writing – review & editing. RD: Conceptualization, Data curation, Formal Analysis, Investigation, Methodology, Software, Visualization, Writing – review & editing. HM: Conceptualization, Data curation, Formal Analysis, Funding acquisition, Investigation, Methodology, Software, Supervision, Visualization, Writing – review & editing. IB-S: Conceptualization, Data curation, Formal Analysis, Investigation, Methodology, Software, Visualization, Writing – review & editing. PL: Conceptualization, Data curation, Formal Analysis, Methodology, Writing – review & editing. TD: Conceptualization, Data curation, Formal Analysis, Methodology, Visualization, Writing – review & editing. IR-R: Conceptualization, Formal Analysis, Funding acquisition, Methodology, Supervision, Writing – review & editing.
