# Supplementary material for: Phenotypic characterization of drought responses in red clover (Trifolium pratense L.)
Source: Front Plant Sci. 2024 Jan 12;14:1304411. doi: 10.3389/fpls.2023.1304411 (PMC10811260; doi:10.3389/fpls.2023.1304411)
Supplement: Supplementary file 1 [file Image_1.pdf]

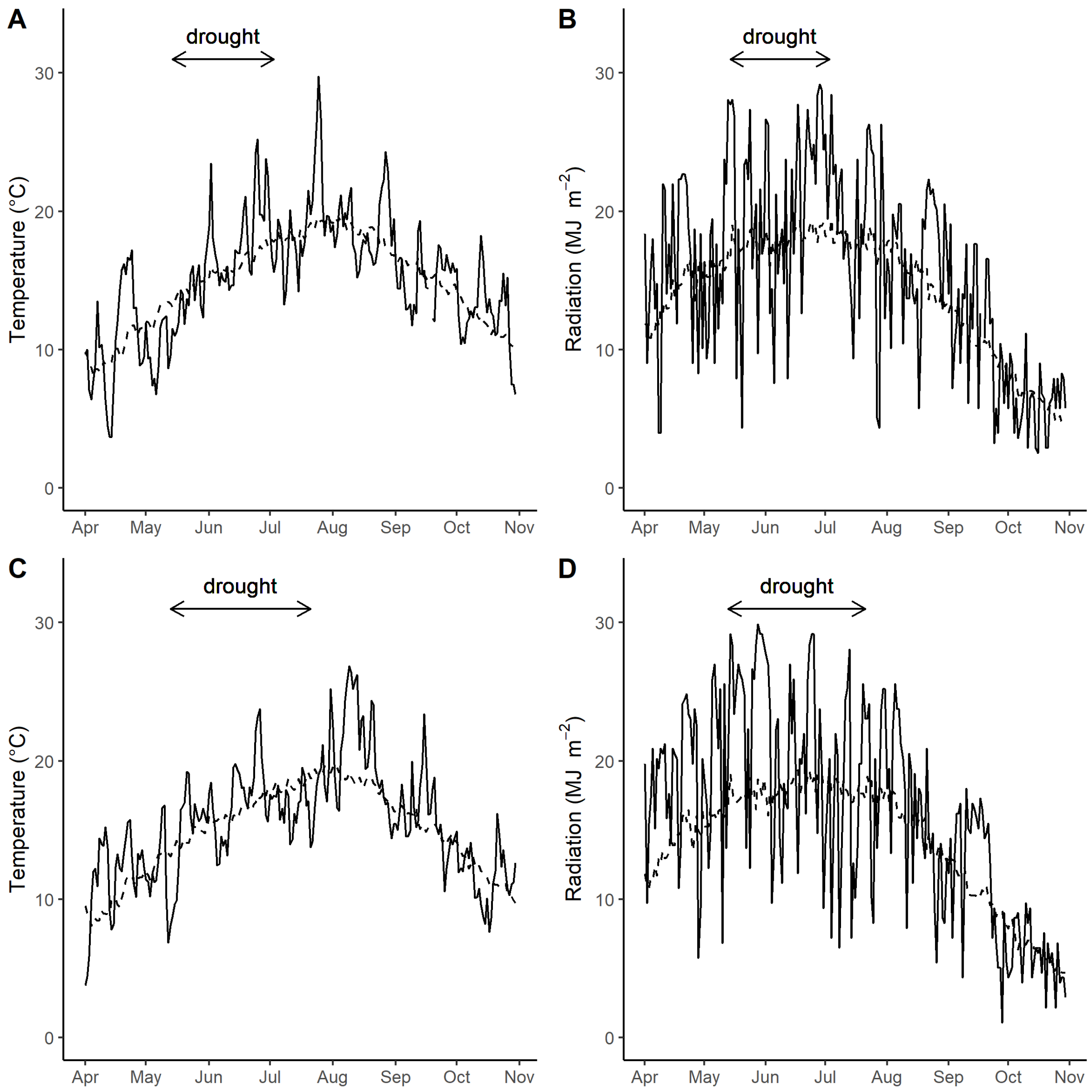

Supplementary Figure S1: Environmental conditions in year 1 (2019) and year 2 (2020): (A) Daily average temperature (full line) in year 1 (A) and year 2 (C) compared to the 30-year average (dashed line); and the sum of daily solar shortwave radiation (full line) in year 1 (B) and year 2 (D) compared to the 30-year average (dashed line). The arrows delineate the period during which the rain-out shelters covered the drought field.
