# Supplementary material for: Phenotypic characterization of drought responses in red clover (Trifolium pratense L.)
Source: Front Plant Sci. 2024 Jan 12;14:1304411. doi: 10.3389/fpls.2023.1304411 (PMC10811260; doi:10.3389/fpls.2023.1304411)
Supplement: Supplementary file 2 [file Image_2.pdf]

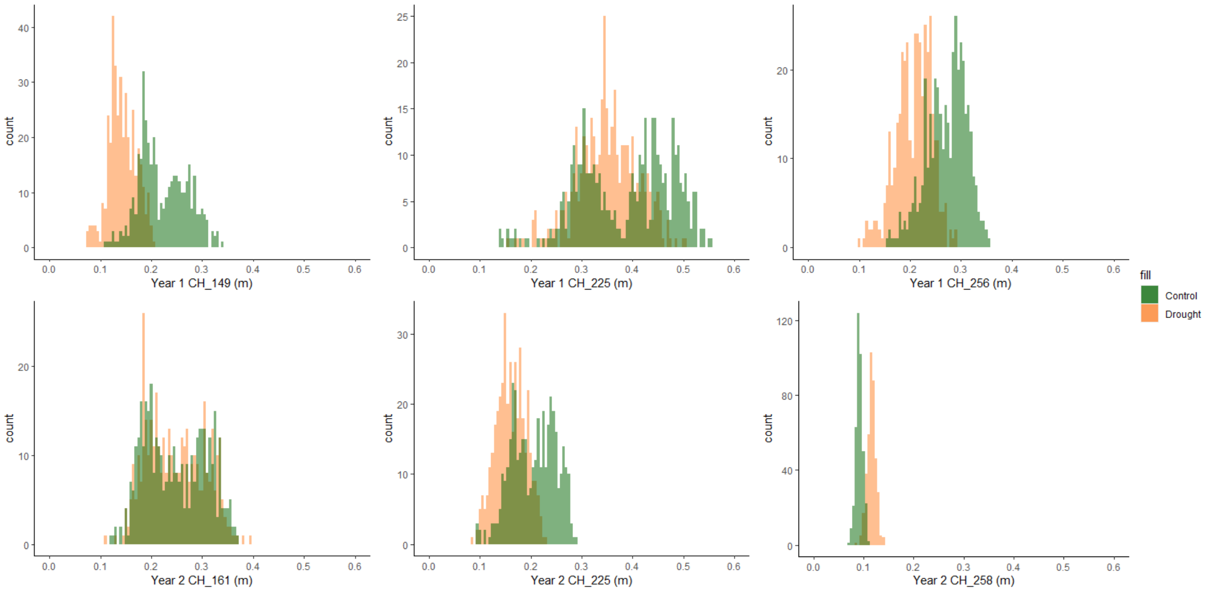


**Supplementary Figure S2: Example of CH data distributions in the growing periods in late spring (left; A and D), summer (middle; B and E), and late summer (right; C and F) of year 1 (top) and year 2 (bottom) in the control field (green) and the drought field (orange). In the control field, CH data appear binomially distributed in late spring and summer, but not in late summer. In the drought field, the effect appears less clear.** Drought treatments were applied between DOY 134 – 184, and DOY 134 – 203 in year 1 and 2, respectively.
