# Supplementary material for: Phenotypic characterization of drought responses in red clover (Trifolium pratense L.)
Source: Front Plant Sci. 2024 Jan 12;14:1304411. doi: 10.3389/fpls.2023.1304411 (PMC10811260; doi:10.3389/fpls.2023.1304411)
Supplement: Supplementary file 3 [file Table_1.docx]

**Supplementary Table S1: Details of the plant material in this study: EUCLEG ID, Name of accession, country of origin, year of cultivar registration or collection, type of material, and collection sites for ecotypes**

| **EUCLEG ID** | **Name of**  **accession** | **Country of**  **Origin or**  **provenance** | **Year of registration**  **or collection** | **Type** | **Collection site** |
| --- | --- | --- | --- | --- | --- |
| EUC_Tp_001 | Dimanche | FRA | 2006 | Cultivar |  |
| EUC_Tp_002 | Discovery | FRA | 2006 | Cultivar |  |
| EUC_Tp_003 | Formica | CHE | 1993 | Cultivar |  |
| EUC_Tp_004 | Milvus | CHE | 1993 | Cultivar |  |
| EUC_Tp_005 | Pavo | CHE | 2002 | Cultivar |  |
| EUC_Tp_006 | S586 AberClaret | GBR | 2005 | Cultivar |  |
| EUC_Tp_007 | S592 AberChianti | GBR | 2006 | Cultivar |  |
| EUC_Tp_008 | Gandalf | NOR | 2015 | Cultivar |  |
| EUC_Tp_009 | Lea | NOR | 2002 | Cultivar |  |
| EUC_Tp_010 | K 17 | SRB | NA | Cultivar |  |
| EUC_Tp_011 | K 38 | SRB | NA | Cultivar |  |
| EUC_Tp_012 | SW Ares | SWE | 2001 | Cultivar |  |
| EUC_Tp_013 | Cyllene | CZE | 2015 | Cultivar |  |
| EUC_Tp_014 | Himalia | CZE | 2012 | Cultivar |  |
| EUC_Tp_015 | Metis | CZE | 2016 | Cultivar |  |
| EUC_Tp_016 | SANGRIA | FRA | 2014 | Cultivar |  |
| EUC_Tp_017 | Saija | FIN | 2006 | Cultivar |  |
| EUC_Tp_018 | Global | BEL | 2002 | Cultivar |  |
| EUC_Tp_019 | Merviot | BEL | 1980 | Cultivar |  |
| EUC_Tp_020 | Bonus | CZE | 2008 | Cultivar |  |
| EUC_Tp_021 | NGB1132 | FIN | 1981 | Landrace | Vanhala, Vihti |
| EUC_Tp_022 | NGB1133 | FIN | 1981 | Landrace | Hailuoto, Hailuoto |
| EUC_Tp_023 | NGB1142 | FIN | 1981 | Landrace | Kotalahti, Leppävirta |
| EUC_Tp_024 | NGB14322 | FIN | 1998 | Ecotype | Roukalahti, Liperi |
| EUC_Tp_025 | NGB1730 | DNK | 1964 | Cultivar |  |
| EUC_Tp_026 | NGB2161 | NOR | NA | Landrace |  |
| EUC_Tp_027 | NGB2391 | SWE | NA | Landrace |  |
| EUC_Tp_028 | NGB2392 | SWE | NA | Landrace |  |
| EUC_Tp_029 | NGB2458 | SWE | NA | Landrace |  |
| EUC_Tp_030 | NGB2461 | SWE | NA | Landrace |  |
| EUC_Tp_031 | NGB2487 | SWE | NA | Landrace |  |
| EUC_Tp_032 | NGB2490 | SWE | NA | Landrace |  |
| EUC_Tp_033 | NGB2492 | SWE | NA | Landrace |  |
| EUC_Tp_034 | NGB4089 | SWE | NA | Landrace |  |
| EUC_Tp_035 | Grasslands Colenso | NZL | 1989 | Cultivar |  |
| EUC_Tp_036 | Sensation | NZL | 2002 | Cultivar |  |
| EUC_Tp_037 | Affoltern i.E._328 | CHE | 1972 | Landrace | Affoltern i.E. |
| EUC_Tp_038 | Affoltern i.E._6 | CHE | 1972 | Landrace | Affoltern i.E. |
| EUC_Tp_039 | Belpberg_225 | CHE | 1972 | Landrace | Belpberg |
| EUC_Tp_040 | Belpberg_226 | CHE | 1972 | Landrace | Belpberg |
| EUC_Tp_041 | Belpberg_229 | CHE | 1972 | Landrace | Belpberg |
| EUC_Tp_042 | Bern_78 | CHE | 1972 | Landrace | Bern |
| EUC_Tp_043 | Bowil_119 | CHE | 1972 | Landrace | Bowil |
| EUC_Tp_044 | Bruetten_3 | CHE | 1972 | Landrace | Bruetten |
| EUC_Tp_045 | Bubikon_8 | CHE | 1972 | Landrace | Bubikon |
| EUC_Tp_046 | Burgistein_300 | CHE | 1972 | Landrace | Burgistein |
| EUC_Tp_047 | Columba | CHE | 2016 | Cultivar |  |
| EUC_Tp_048 | Corvus | CHE | 1995 | Cultivar |  |
| EUC_Tp_049 | Dafila | CHE | 2008 | Cultivar |  |
| EUC_Tp_050 | Frauenkappelen_86 | CHE | 1972 | Landrace | Frauenkappelen |
| EUC_Tp_051 | Goldbach i.E._167 | CHE | 1972 | Landrace | Goldbach |
| EUC_Tp_052 | Huttwil_50 | CHE | 1972 | Landrace | Huttwil |
| EUC_Tp_053 | Huttwil_60 | CHE | 1972 | Landrace | Huttwil |
| EUC_Tp_054 | Koeniz_231 | CHE | 1972 | Landrace | Koeniz |
| EUC_Tp_055 | Koeniz_279 | CHE | 1972 | Landrace | Koeniz |
| EUC_Tp_056 | Krauchthal_176 | CHE | 1972 | Landrace | Krauchthal |
| EUC_Tp_057 | Lanzenhaeusern_291 | CHE | 1972 | Landrace | Lanzenhaeusern |
| EUC_Tp_058 | Lestris | CHE | 2009 | Cultivar |  |
| EUC_Tp_059 | Merula | CHE | 2002 | Cultivar |  |
| EUC_Tp_060 | Milonia | CHE | 2006 | Cultivar |  |
| EUC_Tp_061 | Monaco | CHE | 2012 | Cultivar |  |
| EUC_Tp_062 | Niederwangen_262 | CHE | 1972 | Landrace | Niederwangen |
| EUC_Tp_063 | Niederwangen_75 | CHE | 1972 | Landrace | Niederwangen |
| EUC_Tp_064 | Oberthal_121 | CHE | 1972 | Landrace | Oberthal |
| EUC_Tp_065 | Oberuzwil_280 | CHE | 1972 | Landrace | Oberuzwil |
| EUC_Tp_066 | Pastor | CHE | 2010 | Cultivar |  |
| EUC_Tp_067 | Riedbach_88 | CHE | 1972 | Landrace | Riedbach |
| EUC_Tp_068 | Rueegsau_160 | CHE | 1972 | Landrace | Rueegsau |
| EUC_Tp_069 | Rueti_314 | CHE | 1972 | Landrace | Rueti |
| EUC_Tp_070 | Schmidigen_336 | CHE | 1972 | Landrace | Schmidigen |
| EUC_Tp_071 | Semperina | CHE | 2016 | Cultivar |  |
| EUC_Tp_072 | Signau_140 | CHE | 1972 | Landrace | Signau |
| EUC_Tp_073 | Sumiswald_189 | CHE | 1972 | Landrace | Sumiswald |
| EUC_Tp_074 | Ueberstorf_294 | CHE | 1972 | Landrace | Ueberstorf |
| EUC_Tp_075 | Ueberstorf_346 | CHE | 1972 | Landrace | Ueberstorf |
| EUC_Tp_076 | Ufhusen_52 | CHE | 1972 | Landrace | Ufhusen |
| EUC_Tp_077 | Uttigen_2 | CHE | 1972 | Landrace | Uttigen |
| EUC_Tp_078 | Wynigen_335 | CHE | 1972 | Landrace | Wynigen |
| EUC_Tp_079 | Zaeziwil_125 | CHE | 1972 | Landrace | Zaeziwil |
| EUC_Tp_080 | Zaeziwil_127 | CHE | 1972 | Landrace | Zaeziwil |
| EUC_Tp_081 | Aa 3100 | GBR | 1948 | Cultivar |  |
| EUC_Tp_082 | Aa 3148 | GBR | 1948 | Cultivar |  |
| EUC_Tp_083 | Aa 3149 | GBR | 1948 | Cultivar |  |
| EUC_Tp_084 | AA 32 | GBR | 2011 | Breeding material |  |
| EUC_Tp_085 | Aa 3459 | GBR | 1957 | Cultivar |  |
| EUC_Tp_086 | Aa 4190 | POL | 1990 | Ecotype |  |
| EUC_Tp_087 | Aa 4292 | CZE | 1992 | Ecotype |  |
| EUC_Tp_088 | Aa 4298 | SVK | 1992 | Ecotype |  |
| EUC_Tp_089 | Aa 4351 | BGR | 1993 | Ecotype |  |
| EUC_Tp_090 | Aa 4379 Britta | GBR | 1994 | Cultivar |  |
| EUC_Tp_091 | Aa 4390 | PRT | 1995 | Ecotype |  |
| EUC_Tp_092 | Aa 4400 | GBR | 1996 | Ecotype |  |
| EUC_Tp_093 | Aa 4444 | ITA | 1998 | Ecotype |  |
| EUC_Tp_094 | Aa 4516 | ESP | 2003 | Ecotype |  |
| EUC_Tp_095 | Aa 4519 | ESP | 2003 | Ecotype |  |
| EUC_Tp_097 | Aa 4528 | ESP | 2003 | Ecotype |  |
| EUC_Tp_098 | Aa 4939 | NZL | NA | Cultivar |  |
| EUC_Tp_099 | Aa 5674 | ARG | 2016 | Cultivar |  |
| EUC_Tp_100 | Aa 5675 | ARG | 2016 | Cultivar |  |
| EUC_Tp_101 | Aa 5676 | ARG | 2016 | Cultivar |  |
| EUC_Tp_102 | Aa 5677 | ARG | 2016 | Cultivar |  |
| EUC_Tp_103 | Aa 5678 | ARG | 2016 | Cultivar |  |
| EUC_Tp_104 | Aa 5746 Harmonie | GBR | 2017 | Cultivar |  |
| EUC_Tp_105 | Aa4380 Altaswede | CAN | NA | Cultivar |  |
| EUC_Tp_106 | S543 AberRuby | GBR | NA | Cultivar |  |
| EUC_Tp_107 | Aa 4940 Broadway | NZL | 2003 | Cultivar |  |
| EUC_Tp_108 | TP9525 | CHE | NA | Breeding material |  |
| EUC_Tp_109 | TP9645 | CHE | NA | Breeding material |  |
| EUC_Tp_110 | TP9445 | CHE | NA | Breeding material |  |
| EUC_Tp_111 | TP9735 | CHE | NA | Breeding material |  |
| EUC_Tp_112 | TP9315 | CHE | NA | Breeding material |  |
| EUC_Tp_113 | Gumpensteiner Rotklee | AUT | NA | Cultivar |  |
| EUC_Tp_114 | Cinnamon Plus | USA | NA | Cultivar |  |
| EUC_Tp_115 | DFRC11 | USA | NA | Breeding material |  |
| EUC_Tp_116 | DFRC12 | USA | NA | Breeding material |  |
| EUC_Tp_117 | DFRC13 | USA | NA | Breeding material |  |
| EUC_Tp_118 | DFRC14 | USA | NA | Breeding material |  |
| EUC_Tp_119 | DFRC15 | USA | NA | Breeding material |  |
| EUC_Tp_120 | FF 9615 | USA | NA | Cultivar |  |
| EUC_Tp_121 | Marathon | USA | NA | Cultivar |  |
| EUC_Tp_122 | Starfire I | USA | NA | Cultivar |  |
| EUC_Tp_123 | Starfire II | USA | NA | Cultivar |  |
| EUC_Tp_124 | GnRk0729 | NOR | NA | Breeding material |  |
| EUC_Tp_125 | GnRk0747 | NOR | NA | Breeding material |  |
| EUC_Tp_126 | KvRk0201 | NOR | NA | Breeding material |  |
| EUC_Tp_127 | LGRk8801 | NOR | NA | Breeding material |  |
| EUC_Tp_128 | LGRk9415 | NOR | NA | Breeding material |  |
| EUC_Tp_129 | Linus | NOR | NA | Cultivar |  |
| EUC_Tp_130 | LøRk0286 | NOR | NA | Breeding material |  |
| EUC_Tp_131 | LøRk0287 | NOR | NA | Breeding material |  |
| EUC_Tp_132 | LøRk0390 | NOR | NA | Cultivar |  |
| EUC_Tp_133 | Liv Siri | NOR | 2002 | Cultivar |  |
| EUC_Tp_134 | LøRk9207 | NOR | NA | Breeding material |  |
| EUC_Tp_135 | LøRk9625 | NOR | NA | Breeding material |  |
| EUC_Tp_136 | LøRk9627 | NOR | NA | Breeding material |  |
| EUC_Tp_137 | LøRk9628 | NOR | NA | Breeding material |  |
| EUC_Tp_138 | LøRk9753 | NOR | NA | Breeding material |  |
| EUC_Tp_139 | VåRk0401 | NOR | NA | Breeding material |  |
| EUC_Tp_140 | VåRk0510 | NOR | NA | Breeding material |  |
| EUC_Tp_141 | VåRk0512 | NOR | NA | Breeding material |  |
| EUC_Tp_142 | VåRk0513 | NOR | NA | Breeding material |  |
| EUC_Tp_143 | VåRk0624 | NOR | NA | Breeding material |  |
| EUC_Tp_144 | VåRk0625 | NOR | NA | Breeding material |  |
| EUC_Tp_145 | K 39 | SRB | NA | Cultivar |  |
| EUC_Tp_146 | Diplomat | DEU | NA | Cultivar |  |
| EUC_Tp_147 | SW 1479004 | SWE | NA | Breeding material |  |
| EUC_Tp_148 | SW 1578301 | SWE | NA | Breeding material |  |
| EUC_Tp_149 | SW 1678001 | SWE | NA | Breeding material |  |
| EUC_Tp_150 | SW RK1092 | SWE | NA | Breeding material |  |
| EUC_Tp_151 | SW RK1117 | SWE | NA | Breeding material |  |
| EUC_Tp_152 | SW RK1118 | SWE | NA | Breeding material |  |
| EUC_Tp_153 | SW RK1119 | SWE | NA | Breeding material |  |
| EUC_Tp_154 | SW RK1120 | SWE | NA | Breeding material |  |
| EUC_Tp_155 | SW RK1121 | SWE | NA | Breeding material |  |
| EUC_Tp_156 | SW RK1122 | SWE | NA | Breeding material |  |
| EUC_Tp_157 | SW RK1123 | SWE | NA | Breeding material |  |
| EUC_Tp_158 | SW RK1124 | SWE | NA | Breeding material |  |
| EUC_Tp_159 | SW RK1125 | SWE | NA | Breeding material |  |
| EUC_Tp_160 | SW RK1131 | SWE | NA | Breeding material |  |
| EUC_Tp_161 | SW RK1132 | SWE | NA | Breeding material |  |
| EUC_Tp_162 | SW RK1133 | SWE | NA | Breeding material |  |
| EUC_Tp_163 | SW RK1134 | SWE | NA | Breeding material |  |
| EUC_Tp_164 | SW Yngve | SWE | NA | Cultivar |  |
| EUC_Tp_165 | SWÅ RK09093 | SWE | NA | Breeding material |  |
| EUC_Tp_166 | 0780MP2 | CZE | 2007 | Breeding material |  |
| EUC_Tp_167 | 08102MP2 | CZE | 2008 | Breeding material |  |
| EUC_Tp_168 | 08102MP4 | CZE | 2008 | Breeding material |  |
| EUC_Tp_169 | Callisto | CZE | NA | Cultivar |  |
| EUC_Tp_170 | Elara | CZE | NA | Cultivar |  |
| EUC_Tp_171 | Ganymed | CZE | NA | Cultivar |  |
| EUC_Tp_172 | Hegemon | CZE | NA | Cultivar |  |
| EUC_Tp_173 | Helike | CZE | NA | Cultivar |  |
| EUC_Tp_174 | HŽ 2004 80 - 01 | CZE | 2004 | Breeding material |  |
| EUC_Tp_175 | JL 2n 07 80 MP 1 | CZE | 2007 | Breeding material |  |
| EUC_Tp_176 | Kalyke | CZE | NA | Cultivar |  |
| EUC_Tp_177 | TPD-05-11-18002 | CZE | 2011 | Breeding material |  |
| EUC_Tp_178 | TPD-05-11-3087 | CZE | 2011 | Breeding material |  |
| EUC_Tp_179 | TPD-05-11-3088 | CZE | 2011 | Breeding material |  |
| EUC_Tp_180 | TPD-05-13-3080 | CZE | 2013 | Breeding material |  |
| EUC_Tp_181 | TPD-05-13-3085 | CZE | 2013 | Breeding material |  |
| EUC_Tp_182 | TPD-05-13-3091 | CZE | 2013 | Breeding material |  |
| EUC_Tp_183 | TPD-05-14-1011 | CZE | 2014 | Breeding material |  |
| EUC_Tp_184 | TPD-05-15-3127 | CZE | 2015 | Breeding material |  |
| EUC_Tp_185 | TPD-05-15-3128 | CZE | 2015 | Breeding material |  |
| EUC_Tp_186 | TPD-05-15-3129 | CZE | 2015 | Breeding material |  |
| EUC_Tp_187 | TPD-05-16-3076 | CZE | 2016 | Breeding material |  |
| EUC_Tp_188 | TPD-05-16-3146 | CZE | 2016 | Breeding material |  |
| EUC_Tp_189 | TPD-05-16-3177 | CZE | 2016 | Breeding material |  |
| EUC_Tp_190 | KARIM | FRA | NA | Cultivar |  |
| EUC_Tp_191 | MISTRAL | FRA | NA | Cultivar |  |
| EUC_Tp_192 | RAVVI | FRA | NA | Cultivar |  |
| EUC_Tp_193 | TREVVIO | FRA | NA | Cultivar |  |
| EUC_Tp_194 | Grasslands Hamua | NZL | NA | Cultivar |  |
| EUC_Tp_195 | Grasslands Turoa | NZL | NA | Cultivar |  |
| EUC_Tp_196 | Relish | NZL | NA | Cultivar |  |
| EUC_Tp_197 | Ruby/Enterprise | NZL | NA | Cultivar |  |
| EUC_Tp_198 | Natsuyu | JPN | NA | Cultivar |  |
| EUC_Tp_199 | Ryokuyu | JPN | NA | Cultivar |  |
| EUC_Tp_200 | NS-Mlava | SRB | NA | Cultivar |  |
| EUC_Tp_201 | NS-Petnica | SRB | NA | Cultivar |  |
| EUC_Tp_202 | NS-Sana | SRB | NA | Cultivar |  |
| EUC_Tp_203 | Una(NS) | SRB | NA | Cultivar |  |
| EUC_Tp_204 | Zoja (NS) | SRB | NA | Cultivar |  |
| EUC_Tp_205 | Avisto | BEL | NA | Cultivar |  |
| EUC_Tp_206 | Crossway | NZL | NA | Cultivar |  |
| EUC_Tp_207 | Lemmon | BEL | 2000 | Cultivar |  |
| EUC_Tp_208 | Merkemse | BEL | 1955 | Landrace | Merkem, Belgium |
| EUC_Tp_209 | Tp.12.12 | BEL | NA | Breeding material |  |
| EUC_Tp_210 | Tp.14.7 | BEL | NA | Breeding material |  |
| EUC_Tp_211 | Tp.17.2 | BEL | NA | Cultivar |  |
| EUC_Tp_212 | Agil | CZE | NA | Cultivar |  |
| EUC_Tp_214 | Brisk | CZE | NA | Cultivar |  |
| EUC_Tp_215 | Chlumecký | CZE | NA | Cultivar |  |
| EUC_Tp_218 | Feng | CZE | NA | Cultivar |  |
| EUC_Tp_219 | Garant | CZE | NA | Cultivar |  |
| EUC_Tp_223 | Respect | CZE | NA | Cultivar |  |
| EUC_Tp_224 | Slavín | CZE | NA | Cultivar |  |
| EUC_Tp_225 | Slavoj | CZE | NA | Cultivar |  |
| EUC_Tp_227 | Spurt | CZE | NA | Cultivar |  |
| EUC_Tp_228 | Start | CZE | NA | Cultivar |  |
| EUC_Tp_229 | Suez | CZE | NA | Cultivar |  |
| EUC_Tp_231 | Trubadur | CZE | NA | Cultivar |  |
| EUC_Tp_232 | Van | CZE | NA | Cultivar |  |
| EUC_Tp_233 | Vendelín | CZE | NA | Cultivar |  |
| EUC_Tp_234 | Vltavín | CZE | NA | Cultivar |  |
| EUC_Tp_236 | Zefyr | CZE | NA | Cultivar |  |
| EUC_Tp_237 | Harmonie | DEU | NA | Cultivar |  |
| EUC_Tp_238 | Regent | DEU | NA | Cultivar |  |
| EUC_Tp_239 | NGB1736 | DNK | NA | Cultivar |  |
| EUC_Tp_240 | NGB2347 | SWE | NA | Cultivar |  |
| EUC_Tp_241 | NGB2349 | SWE | NA | Cultivar |  |
| EUC_Tp_242 | NGB2395 | SWE | NA | Landrace |  |
| EUC_Tp_243 | NGB2452 | SWE | NA | Landrace |  |
| EUC_Tp_244 | NGB2453 | SWE | NA | Landrace |  |
| EUC_Tp_245 | NGB2464 | SWE | NA | Landrace |  |
| EUC_Tp_246 | NGB2465 | SWE | NA | Landrace |  |
| EUC_Tp_247 | NGB2466 | SWE | NA | Landrace |  |
| EUC_Tp_248 | NGB2468 | SWE | NA | Landrace |  |
| EUC_Tp_249 | NGB2469 | SWE | NA | Landrace |  |
| EUC_Tp_250 | NGB2471 | SWE | NA | Landrace |  |
| EUC_Tp_251 | NGB2472 | SWE | NA | Landrace |  |
| EUC_Tp_252 | NGB2473 | SWE | NA | Landrace |  |
| EUC_Tp_253 | NGB2474 | SWE | NA | Landrace |  |
| EUC_Tp_254 | NGB2475 | SWE | NA | Landrace |  |
| EUC_Tp_255 | NGB2476 | SWE | NA | Landrace |  |
| EUC_Tp_256 | NGB2477 | SWE | NA | Landrace |  |
| EUC_Tp_257 | NGB2481 | SWE | NA | Landrace |  |
| EUC_Tp_258 | NGB2482 | SWE | NA | Landrace |  |
| EUC_Tp_259 | NGB2494 | SWE | NA | Landrace |  |
| EUC_Tp_260 | NGB2495 | SWE | NA | Landrace |  |
| EUC_Tp_261 | NGB2569 | SWE | NA | Landrace |  |
| EUC_Tp_262 | NGB2598 | SWE | NA | Landrace |  |
| EUC_Tp_263 | NGB2599 | SWE | NA | Landrace |  |
| EUC_Tp_264 | NGB2600 | SWE | NA | Landrace |  |
| EUC_Tp_265 | NGB2739 | SWE | NA | Cultivar |  |
| EUC_Tp_266 | NGB2740 | SWE | NA | Cultivar |  |
| EUC_Tp_267 | NGB2742 | SWE | NA | Cultivar |  |
| EUC_Tp_268 | NGB2745 | SWE | NA | Cultivar |  |
| EUC_Tp_269 | NGB2746 | SWE | NA | Cultivar |  |
| EUC_Tp_270 | NGB2747 | SWE | NA | Cultivar |  |
| EUC_Tp_271 | NGB2748 | SWE | NA | Cultivar |  |
| EUC_Tp_272 | NGB2749 | SWE | NA | Cultivar |  |
| EUC_Tp_273 | NGB2750 | SWE | NA | Cultivar |  |
| EUC_Tp_274 | NGB2751 | SWE | NA | Cultivar |  |
| EUC_Tp_275 | NGB4126 | DNK | NA | Cultivar |  |
| EUC_Tp_277 | NGB7510 | SWE | NA | Cultivar |  |
| EUC_Tp_278 | NGB9966 | SWE | NA | Landrace |  |
| EUC_Tp_279 | Diadem | FRA | NA | Cultivar |  |
| EUC_Tp_280 | Diper | FRA | NA | Cultivar |  |
| EUC_Tp_281 | Diplo | FRA | NA | Cultivar |  |
| EUC_Tp_282 | Kindia | FRA | NA | Cultivar |  |
| EUC_Tp_283 | Affoltern i.E._325 | CHE | 1972 | Landrace | Affoltern i.E. |
| EUC_Tp_284 | Bern_76 | CHE | 1972 | Landrace | Bern |
| EUC_Tp_285 | Bigenthal_163 | CHE | 1972 | Landrace | Bigenthal |
| EUC_Tp_286 | Biglen_352 | CHE | 1972 | Landrace | Biglen |
| EUC_Tp_287 | Englisberg_249 | CHE | 1972 | Landrace | Englisberg |
| EUC_Tp_288 | Grossdietwil_21 | CHE | 1972 | Landrace | Grossdietwil |
| EUC_Tp_289 | Haeusernmoos_333 | CHE | 1972 | Landrace | Haeusernmoos |
| EUC_Tp_290 | Koeniz_239 | CHE | 1972 | Landrace | Koeniz |
| EUC_Tp_291 | Koeniz_247 | CHE | 1972 | Landrace | Koeniz |
| EUC_Tp_292 | Lauperswil_138 | CHE | 1972 | Landrace | Lauperswil |
| EUC_Tp_293 | MontCalme | CHE | NA | Cultivar |  |
| EUC_Tp_294 | Neuenegg_340 | CHE | 1972 | Landrace | Neuenegg |
| EUC_Tp_295 | Niederscherli_273 | CHE | 1972 | Landrace | Niederscherli |
| EUC_Tp_296 | Oberbottigen_7 | CHE | 1972 | Landrace | Oberbottigen |
| EUC_Tp_297 | Oberoenz_321 | CHE | 1972 | Landrace | Oberoenz |
| EUC_Tp_298 | Oeschenbach_330 | CHE | 1972 | Landrace | Oeschenbach |
| EUC_Tp_299 | Renova | CHE | NA | Cultivar |  |
| EUC_Tp_300 | Riggisberg_318 | CHE | 1972 | Landrace | Riggisberg |
| EUC_Tp_301 | Rueedisbach_332 | CHE | 1972 | Landrace | Rueedisbach |
| EUC_Tp_302 | Rüttinova | CHE | NA | Cultivar |  |
| EUC_Tp_303 | Schmitten_5 | CHE | 1972 | Landrace | Schmitten |
| EUC_Tp_304 | Signau_154 | CHE | 1972 | Landrace | Signau |
| EUC_Tp_305 | Wasen i.E._199 | CHE | 1972 | Landrace | Wasen i.E. |
| EUC_Tp_306 | Weier i.E._327 | CHE | 1972 | Landrace | Weier i.E. |
| EUC_Tp_307 | Aa 3090 | GBR | 1948 | Cultivar |  |
| EUC_Tp_308 | Aa 3108 | GBR | 1948 | Cultivar |  |
| EUC_Tp_309 | Aa 3507 | IRN | 1957 | Ecotype | Iran, farmland |
| EUC_Tp_310 | Aa 4189 | POL | 1990 | Ecotype |  |
| EUC_Tp_311 | Aa 4297 | CZE | 1992 | Ecotype |  |
| EUC_Tp_312 | Aa 4398 | GBR | 1996 | Ecotype |  |
| EUC_Tp_313 | Aa 4403 | GBR | 1996 | Ecotype |  |
| EUC_Tp_315 | Aa 4445 | ITA | 1998 | Ecotype |  |
| EUC_Tp_316 | Aa 4448 | ITA | 1998 | Ecotype |  |
| EUC_Tp_317 | Aa 4456 | ITA | 1998 | Ecotype |  |
| EUC_Tp_318 | Aa 4515 | ESP | 2003 | Ecotype |  |
| EUC_Tp_319 | Aa 4520 | ESP | 2005 | Ecotype |  |
| EUC_Tp_320 | Aa 4525 | ESP | 2003 | Ecotype |  |
| EUC_Tp_321 | Aa 4527 | ESP | 2003 | Ecotype |  |
| EUC_Tp_322 | Aa 4529 | ESP | 2003 | Ecotype |  |
| EUC_Tp_324 | Aa 4593 | GBR | 2009 | Landrace |  |
| EUC_Tp_325 | Aa 4934 | GBR | 2012 | Breeding material |  |
| EUC_Tp_326 | Aa 4936 | GBR | 2012 | Breeding material |  |
| EUC_Tp_327 | Aa 4937 | GBR | 2012 | Breeding material |  |
| EUC_Tp_328 | Aa 5417 | GBR | 2015 | Breeding material |  |
| EUC_Tp_329 | SW 1479001 | SWE | NA | Breeding material |  |
| EUC_Tp_330 | SW 1479002 | SWE | NA | Breeding material |  |
| EUC_Tp_331 | SW 1479003 | SWE | NA | Breeding material |  |
| EUC_Tp_332 | SW 1678002 | SWE | NA | Breeding material |  |
| EUC_Tp_333 | SW 1678003 | SWE | NA | Breeding material |  |
| EUC_Tp_334 | SW 1678004 | SWE | NA | Breeding material |  |
| EUC_Tp_335 | SW 1678401 | SWE | NA | Breeding material |  |
| EUC_Tp_336 | SW 1678402 | SWE | NA | Breeding material |  |
| EUC_Tp_337 | SW 1678403 | SWE | NA | Breeding material |  |
| EUC_Tp_338 | SW RK1095 | SWE | NA | Breeding material |  |
| EUC_Tp_339 | SW RK1096 | SWE | NA | Breeding material |  |
| EUC_Tp_340 | SW RK1097 | SWE | NA | Breeding material |  |
| EUC_Tp_341 | SW RK1102 | SWE | NA | Breeding material |  |
| EUC_Tp_342 | SW RK1160 | SWE | NA | Breeding material |  |
| EUC_Tp_343 | SW RK1161 | SWE | NA | Breeding material |  |
| EUC_Tp_344 | SW RK1162 | SWE | NA | Breeding material |  |
| EUC_Tp_345 | SW RK1164 | SWE | NA | Breeding material |  |
| EUC_Tp_346 | SWA 1376104 | SWE | NA | Breeding material |  |
| EUC_Tp_347 | SWA 1376105 | SWE | NA | Breeding material |  |
| EUC_Tp_348 | SWA 1476014 | SWE | NA | Breeding material |  |
| EUC_Tp_349 | SWA 1476016 | SWE | NA | Breeding material |  |
| EUC_Tp_350 | SWA 1476017 | SWE | NA | Breeding material |  |
| EUC_Tp_351 | SWA 1476019 | SWE | NA | Breeding material |  |
| EUC_Tp_352 | SWA 1575301 | SWE | NA | Breeding material |  |
| EUC_Tp_353 | SWA 1575302 | SWE | NA | Breeding material |  |
| EUC_Tp_354 | SWA 1575304 | SWE | NA | Breeding material |  |
| EUC_Tp_355 | SWA 1575305 | SWE | NA | Breeding material |  |
| EUC_Tp_356 | SWA 1575306 | SWE | NA | Breeding material |  |
| EUC_Tp_357 | SWA 1575307 | SWE | NA | Breeding material |  |
| EUC_Tp_358 | SWA 1575308 | SWE | NA | Breeding material |  |
| EUC_Tp_359 | SWA 1575309 | SWE | NA | Breeding material |  |
| EUC_Tp_360 | SWA 1575312 | SWE | NA | Breeding material |  |
| EUC_Tp_361 | SWA 1576005 | SWE | NA | Breeding material |  |
| EUC_Tp_362 | SWA 1675205 | SWE | NA | Breeding material |  |
| EUC_Tp_363 | SWA 1675206 | SWE | NA | Breeding material |  |
| EUC_Tp_364 | SWA 1675207 | SWE | NA | Breeding material |  |
| EUC_Tp_365 | SWA 1675208 | SWE | NA | Breeding material |  |
| EUC_Tp_366 | SWA 1675212 | SWE | NA | Breeding material |  |
| EUC_Tp_367 | TPD-05-04-3000 | CZE | 2004 | Breeding material |  |
| EUC_Tp_368 | TPD-05-11-3007 | CZE | 2011 | Breeding material |  |
| EUC_Tp_369 | TPD-05-12-3018 | CZE | 2012 | Breeding material |  |
| EUC_Tp_370 | Avala (NS) | SRB | NA | Cultivar |  |
| EUC_Tp_371 | BL-1-Banja Luka | SRB | 1996 | Breeding material |  |
| EUC_Tp_372 | BL-3-Banja Luka | SRB | 1996 | Breeding material |  |
| EUC_Tp_373 | BL-4-Banja Luka | SRB | 1996 | Breeding material |  |
| EUC_Tp_374 | BL-5-Banja Luka | SRB | 1996 | Breeding material |  |
| EUC_Tp_375 | D-1 | SRB | 2008 | Breeding material |  |
| EUC_Tp_376 | D-10 | SRB | 2008 | Breeding material |  |
| EUC_Tp_377 | D-2 | SRB | 2008 | Breeding material |  |
| EUC_Tp_378 | D-3 | SRB | 2008 | Breeding material |  |
| EUC_Tp_379 | D-4 | SRB | 2008 | Breeding material |  |
| EUC_Tp_380 | D-5 | SRB | 2008 | Breeding material |  |
| EUC_Tp_381 | D-6 | SRB | 2008 | Breeding material |  |
| EUC_Tp_382 | D-7 | SRB | 2008 | Breeding material |  |
| EUC_Tp_383 | D-8 | SRB | 2008 | Breeding material |  |
| EUC_Tp_384 | D-9 | SRB | 2008 | Breeding material |  |
| EUC_Tp_385 | M10-Kopaonik | SRB | 1996 | Ecotype |  |
| EUC_Tp_386 | M11-Kopaonik | SRB | 1996 | Ecotype |  |
| EUC_Tp_387 | M12-Kopaonik | SRB | 1996 | Ecotype |  |
| EUC_Tp_388 | M13-Kopaonik | SRB | 1996 | Ecotype |  |
| EUC_Tp_389 | M14-Kopaonik | SRB | 1996 | Ecotype |  |
| EUC_Tp_390 | NS-Ravanica | SRB | NA | Cultivar |  |
| EUC_Tp_391 | Broadway | NZL | 2003 | Cultivar |  |
| EUC_Tp_392 | Kontiki | DEU | NA | Cultivar |  |
| EUC_Tp_393 | Mercury | BEL | NA | Cultivar |  |
| EUC_Tp_394 | Merian | BEL | 2000 | Cultivar |  |
| EUC_Tp_395 | Oudenaerdse | BEL | 1950 | Landrace | Oudenaarde, Belgium |
| EUC_Tp_396 | Primus | BEL | 1967 | Landrace | unspecified location, Belgium |
| EUC_Tp_397 | Tp.08.4 | BEL | NA | Breeding material |  |
| EUC_Tp_398 | Tp.08.5 | BEL | NA | Breeding material |  |
| EUC_Tp_399 | Violetta | BEL | NA | Cultivar |  |
| EUC_Tp_400 | Waesse | BEL | 1950 | Landrace | Waasland, Belgium |
| EUC_Tp_446 | Affoltern i.E._186 | CHE | 1972 | Landrace | Affoltern i.E. |
| EUC_Tp_447 | Arni b.Biglen_351 | CHE | 1972 | Landrace | Arni bei Biglen |
| EUC_Tp_449 | Horgen_1 | CHE | 1972 | Landrace | Horgen |
| EUC_Tp_454 | Lanzenhaeusern_292 | CHE | 1972 | Landrace | Lanzenhaeusern |
| EUC_Tp_456 | Riggisberg_311 | CHE | 1972 | Landrace | Riggisberg |
| EUC_Tp_660 | LøRk0498 | NOR | NA | Breeding material |  |
| EUC_Tp_661 | SWA 1575303 | SWE | NA | Breeding material |  |
| EUC_Tp_662 | SWA 1576001 | SWE | NA | Breeding material |  |
| CONTROL | Lemmon | BEL | 2000 | Cultivar |  |
