# Supplementary material for: Phenotypic characterization of drought responses in red clover (Trifolium pratense L.)
Source: Front Plant Sci. 2024 Jan 12;14:1304411. doi: 10.3389/fpls.2023.1304411 (PMC10811260; doi:10.3389/fpls.2023.1304411)
Supplement: Supplementary file 4 [file Table_2.docx]

**Supplementary Table S2: For every variable in this study the number of plots (out of 795 total plots in the trial) retained for analysis, the type of mixed model used to calculate the accession averages, and the broad-sense heritability.**

| **Year** | **Variable** | **Control field** | | |  | **Drought field** | | |
| --- | --- | --- | --- | --- | --- | --- | --- | --- |
|  |  | **# Plots included** | **Model used** | **Heritability**  **(H²)** |  | **# Plots included** | **Model used** | **Heritability**  **(H²)** |
| 2019 | FLD | 788 | 2 | 0.72 |  | 790 | 1 | 0.71 |
| 2019 | CC_133 | 788 | 2 | 0.24 |  | 790 | 6 | 0.22 |
| 2019 | CC_149 | 788 | 5 | 0.50 |  | 790 | 6 | 0.37 |
| 2019 | CC_165 | 788 | 6 | 0.18 |  | 790 | 6 | 0.17 |
| 2019 | CC_178 | 788 | 3 | 0.27 |  | 731 | 6 | 0.51 |
| 2019 | CC_198 | 788 | 6 | 0.58 |  | 789 | 2 | 0.35 |
| 2019 | CC_205 | 788 | 6 | 0.50 |  | 790 | 2 | 0.33 |
| 2019 | CC_218 | 788 | 6 | 0.27 |  | 790 | 5 | 0.30 |
| 2019 | CC_225 | 788 | 6 | 0.46 |  | 790 | 5 | 0.33 |
| 2019 | CC_238 | 788 | 6 | 0.62 |  | 790 | 5 | 0.63 |
| 2019 | CC_245 | 788 | 6 | 0.52 |  | 790 | 5 | 0.42 |
| 2019 | CC_256 | 788 | 6 | 0.44 |  | 790 | 5 | 0.48 |
| 2019 | CC_262 | 788 | 6 | 0.45 |  | 790 | 5 | 0.48 |
| 2019 | CC_273 | 788 | 6 | 0.56 |  | 790 | 5 | 0.50 |
| 2019 | CC_280 | 788 | 6 | 0.62 |  | 790 | 6 | 0.61 |
| 2019 | CC_288 | 788 | 6 | 0.54 |  | 790 | 3 | 0.43 |
| 2019 | CC_295 | 788 | 6 | 0.55 |  | 790 | 3 | 0.37 |
| 2019 | CH_133 | 788 | 5 | 0.55 |  | 790 | 6 | 0.53 |
| 2019 | CH_149 | 788 | 5 | 0.80 |  | 790 | 6 | 0.62 |
| 2019 | CH_165 | 788 | 5 | 0.84 |  | 790 | 3 | 0.52 |
| 2019 | CH_178 | 788 | 3 | 0.17 |  | 731 | 6 | 0.47 |
| 2019 | CH_198 | 788 | 6 | 0.51 |  | 789 | 6 | 0.26 |
| 2019 | CH_205 | 788 | 6 | 0.75 |  | 790 | 6 | 0.46 |
| 2019 | CH_218 | 788 | 6 | 0.79 |  | 790 | 3 | 0.51 |
| 2019 | CH_225 | 788 | 6 | 0.84 |  | 790 | 3 | 0.50 |
| 2019 | CH_238 | 788 | 6 | 0.54 |  | 790 | 6 | 0.43 |
| 2019 | CH_245 | 788 | 6 | 0.67 |  | 790 | 5 | 0.51 |
| 2019 | CH_256 | 788 | 6 | 0.76 |  | 790 | 6 | 0.71 |
| 2019 | CH_262 | 788 | 6 | 0.78 |  | 790 | 6 | 0.72 |
| 2019 | CH_273 | 788 | 6 | 0.82 |  | 790 | 6 | 0.70 |
| 2019 | CH_280 | 788 | 6 | 0.12 |  | 790 | 6 | 0.00 |
| 2019 | CH_288 | 788 | 6 | 0.42 |  | 790 | 6 | 0.23 |
| 2019 | CH_295 | 788 | 6 | 0.71 |  | 790 | 6 | 0.45 |
| 2019 | CH_man_151 | 785 | 6 | 0.52 |  | 788 | 6 | 0.57 |
| 2019 | CH_man_171 | 788 | 5 | 0.78 |  | 731 | 3 | 0.59 |
| 2019 | CH_man_183 | 0 | NA | NA |  | 679 | 3 | 0.47 |
| 2019 | CH_man_225 | 788 | 6 | 0.83 |  | 790 | 3 | 0.63 |
| 2019 | CH_man_270 | 788 | 1 | 0.63 |  | 790 | 6 | 0.70 |
| 2019 | CWSI_206 | 788 | 6 | 0.5 |  | 790 | 6 | 0.23 |
| 2020 | CC_127 | 788 | 4 | 0.12 |  | 790 | 3 | 0.18 |
| 2020 | CC_139 | 787 | 5 | 0.54 |  | 790 | 3 | 0.25 |
| 2020 | CC_155 | 788 | 5 | 0.43 |  | 790 | 6 | 0.38 |
| 2020 | CC_161 | 788 | 5 | 0.38 |  | 790 | 6 | 0.35 |
| 2020 | CC_175 | 788 | 2 | 0.20 |  | 790 | 3 | 0.37 |
| 2020 | CC_189 | 788 | 3 | 0.16 |  | 790 | 5 | 0.33 |
| 2020 | CC_195 | 788 | 3 | 0.13 |  | 790 | 6 | 0.37 |
| 2020 | CC_204 | 787 | 4 | 0.41 |  | 776 | 3 | 0.24 |
| 2020 | CC_212 | 788 | 5 | 0.43 |  | 790 | 6 | 0.31 |
| 2020 | CC_220 | 788 | 5 | 0.38 |  | 790 | 6 | 0.28 |
| 2020 | CC_225 | 788 | 5 | 0.37 |  | 790 | 6 | 0.23 |
| 2020 | CC_245 | 787 | 3 | 0.45 |  | 789 | 3 | 0.18 |
| 2020 | CC_258 | 787 | 3 | 0.35 |  | 789 | 3 | 0.32 |
| 2020 | CC_266 | 787 | 2 | 0.28 |  | 789 | 6 | 0.25 |
| 2020 | CC_288 | 787 | 2 | 0.33 |  | 789 | 6 | 0.29 |
| 2020 | CH_127 | 788 | 5 | 0.57 |  | 790 | 6 | 0.50 |
| 2020 | CH_139 | 786 | 6 | 0.41 |  | 783 | 3 | 0.38 |
| 2020 | CH_155 | 788 | 6 | 0.65 |  | 790 | 3 | 0.61 |
| 2020 | CH_161 | 788 | 6 | 0.66 |  | 790 | 3 | 0.61 |
| 2020 | CH_175 | 788 | 6 | 0.67 |  | 790 | 3 | 0.56 |
| 2020 | CH_189 | 788 | 6 | 0.27 |  | 790 | 3 | 0.46 |
| 2020 | CH_195 | 788 | 6 | 0.25 |  | 790 | 3 | 0.41 |
| 2020 | CH_204 | 785 | 5 | 0.15 |  | 771 | 3 | 0.20 |
| 2020 | CH_212 | 788 | 6 | 0.56 |  | 790 | 6 | 0.42 |
| 2020 | CH_220 | 788 | 5 | 0.59 |  | 790 | 6 | 0.46 |
| 2020 | CH_225 | 788 | 6 | 0.58 |  | 790 | 6 | 0.49 |
| 2020 | CH_245 | 788 | 5 | 0.63 |  | 789 | 6 | 0.57 |
| 2020 | CH_258 | 788 | 6 | 0.27 |  | 789 | 6 | 0.16 |
| 2020 | CH_266 | 788 | 6 | 0.43 |  | 789 | 6 | 0.34 |
| 2020 | CH_288 | 788 | 5 | 0.42 |  | 789 | 3 | 0.44 |
| 2020 | CH_man_134 | 788 | 5 | 0.52 |  | 790 | 6 | 0.49 |
| 2020 | CH_man_153 | 788 | 6 | 0.57 |  | 789 | 3 | 0.47 |
| 2020 | CH_man_168 | 788 | 5 | 0.68 |  | 790 | 3 | 0.61 |
| 2020 | CH_man_185 | 0 | NA | NA |  | 790 | 3 | 0.52 |
| 2020 | CH_man_240 | 787 | 6 | 0.68 |  | 785 | 6 | 0.57 |
| 2020 | CH_man_288 | 784 | 6 | 0.42 |  | 787 | 6 | 0.41 |
| 2020 | CWSI_194 | 788 | 3 | 0.13 |  | 790 | 2 | 0.26 |
