# Supplementary material for: Phenotypic characterization of drought responses in red clover (Trifolium pratense L.)
Source: Front Plant Sci. 2024 Jan 12;14:1304411. doi: 10.3389/fpls.2023.1304411 (PMC10811260; doi:10.3389/fpls.2023.1304411)
Supplement: Supplementary file 5 [file Table_3.docx]

**Supplementary Table S3: Pearson correlation coefficients over all accessions between ground measured observations for CH and UAV-derived measurements for CH in the drought and control fields in (A) year 1, and (B) year 2.**

**A**: year 1

| **Variable** | **Field** | **CH_149** | **CH_165** | **CH_178** | **CH_225** | **CH_262** | **CH_273** |
| --- | --- | --- | --- | --- | --- | --- | --- |
| CH_man_151 | control | 0.84 |  |  |  |  |  |
|  | drought | 0.85 |  |  |  |  |  |
| CH_man_171 | control |  | 0.91 |  |  |  |  |
|  | drought |  | 0.86 |  |  |  |  |
| CH_man_183 | control |  |  | NA |  |  |  |
|  | drought |  |  | 0.85 |  |  |  |
| CH_man_225 | control |  |  |  | 0.93 |  |  |
|  | drought |  |  |  | 0.87 |  |  |
| CH_man_270 | control |  |  |  |  | 0.83 | 0.82 |
|  | drought |  |  |  |  | 0.90 | 0.90 |

**B**: year 2

| **Variable** | **Field** | **CH_127** | **CH_155** | **CH_161** | **CH_175** | **CH_189** | **CH_245** | **CH_288** |
| --- | --- | --- | --- | --- | --- | --- | --- | --- |
| CH_man_134 | control | 0.94 |  |  |  |  |  |  |
|  | drought | 0.94 |  |  |  |  |  |  |
| CH_man_153 | control |  | 0.94 |  |  |  |  |  |
|  | drought |  | 0.86 |  |  |  |  |  |
| CH_man_168 | control |  |  | 0.97 | 0.93 |  |  |  |
|  | drought |  |  | 0.95 | 0.92 |  |  |  |
| CH_man_185 | control |  |  |  |  | NA |  |  |
|  | drought |  |  |  |  | 0.88 |  |  |
| CH_man_240 | control |  |  |  |  |  | 0.88 |  |
|  | drought |  |  |  |  |  | 0.92 |  |
| CH_man_288 | control |  |  |  |  |  |  | 0.80 |
|  | drought |  |  |  |  |  |  | 0.87 |
